# Safety and tolerability of spermidine supplementation in mice and older adults with subjective cognitive decline

**DOI:** 10.18632/aging.101354

**Published:** 2018-01-08

**Authors:** Claudia Schwarz, Slaven Stekovic, Miranka Wirth, Gloria Benson, Philipp Royer, Stephan J Sigrist, Thomas Pieber, Christopher Dammbrueck, Christoph Magnes, Tobias Eisenberg, Tobias Pendl, Jens Bohlken, Theresa Köbe, Frank Madeo, Agnes Flöel

**Affiliations:** 1 Charité - Universitätsmedizin Berlin, Corporate Member of Freie Universität Berlin, Humboldt-Universität zu Berlin, and Berlin Institute of Health, Klinik und Hochschulambulanz für Neurologie, Berlin, Germany; 2 Charité - Universitätsmedizin Berlin, Corporate Member of Freie Universität Berlin, Humboldt-Universität zu Berlin, and Berlin Institute of Health, NeuroCure Cluster of Excellence, Berlin, Germany; 3 Institute of Molecular Biosciences, University of Graz, NAWI Graz, Graz, Austria; 4 Charité - Universitätsmedizin Berlin, Corporate Member of Freie Universität Berlin, Humboldt-Universität zu Berlin, and Berlin Institute of Health, Center for Stroke Research Berlin, Berlin, Germany; 5 Institute for Biology/Genetics, Freie Universität Berlin, Berlin, Germany; 6 BioTechMed, Graz, Austria; 7 Department of Internal Medicine, Medical University of Graz, Graz, Austria; 8 Joanneum Research Forschungsgesellschaft m.b.H., HEALTH, Institute for Biomedicine and Health Sciences, Graz, Austria; 9 Medical Practice Bohlken for Neurology and Psychiatry, Berlin, Germany; 10 Department of Neurology, University Medicine Greifswald, Greifswald, Germany; * Equal contribution

**Keywords:** polyamines, dietary supplement, spermidine, safety, subjective cognitive decline, aging

## Abstract

Supplementation of spermidine, an autophagy-inducing agent, has been shown to protect against neurodegeneration and cognitive decline in aged animal models. The present translational study aimed to determine safety and tolerability of a wheat germ extract containing enhanced spermidine concentrations. In a preclinical toxicity study, supplementation of spermidine using this extract did not result in morbidities or changes in behavior in BALBc/Rj mice during the 28-days repeated-dose tolerance study. Post mortem examination of the mice organs showed no increase in tumorigenic and fibrotic events. In the human cohort (participants with subjective cognitive decline, n=30, 60 to 80 years of age), a 3-month randomized, placebo-controlled, double-blind Phase II trial was conducted with supplementation of the spermidine-rich plant extract (dosage: 1.2 mg/day). No differences were observed between spermidine and placebo-treated groups in vital signs, weight, clinical chemistry and hematological parameters of safety, as well as in self-reported health status at the end of intervention. Compliance rates above 85% indicated excellent tolerability. The data demonstrate that spermidine supplementation using a spermidine-rich plant extract is safe and well-tolerated in mice and older adults. These findings allow for longer-term intervention studies in humans to investigate the impact of spermidine treatment on cognition and brain integrity.

## Introduction

The natural and ubiquitously occurring polyamines (spermidine, spermine) and the diamine putrescine, result from amino acid metabolism and comprise essential cellular functions, including regulation of cell growth, proliferation and autophagy [1]. Concentration of polyamines in the body is sustained by endogenous biosynthesis, microbial activity in the intestines, and exogenous food intake. However, it has been shown that intracellular polyamine concentrations of several organs decline with age in animals [2] and humans [3-6].

Previous studies indicate that higher intake of polyamines could represent a feasible approach to restore endogenous polyamine concentrations, enhance autophagy rates, and possibly improve health in aging organisms. A translational study, for example, showed that higher external supply of dietary polyamines, administered via a polyamine-enriched diet in mice (26 weeks) and humans (two months), increased blood spermine concentration in both rodents and healthy middle aged males [7]. In aging fruit flies, spermidine-rich feeding inhibited the development of age-dependent memory impairment by restoring polyamine levels in the brain of aging flies and enhancing autophagy [8]. Moreover, increased external administration of polyamines (spermidine in particular) is suggested to promote longevity and autophagy in worms, flies, yeast, and mice [9,10].

The above-described benefits suggest dietary supplementation with spermidine as a promising prevention strategy in older adults with an elevated risk of developing dementia. However, before conducting larger studies with cognitive outcomes in human trials, safety and tolerability of polyamine supplementation need to be established. Based on higher levels of polyamines in some tumors and positive regulation of cell growth and proliferation by polyamines, poly-amines have been indicated as potential enhancers of tumorigenesis [11]. However, tumor frequencies of physiologically aging C57BL6 wild type mice remained unchanged even after life-long dietary supplementation of spermidine [12]. A recent study further demonstrated that oral spermidine administration reduced the incidence of chemically-induced hepatocellular carcinoma and liver fibrosis in mice and caused a life span extension of 25% [13]. In addition, spermidine and other caloric restriction mimetics were shown to induce anticancer immune-surveillance in mice [14], which supports previous observation that higher polyamine intake inhibits the emergence of tumors in rodents, while promoting growth of existing ones [15]. The only study conducted so far on the effect of polyamine-rich diet in men [7] reported no adverse events (AEs) with external supply of polyamines. However, detailed safety assessments were not conducted in this study.

The present study aimed to determine safety and tolerability of spermidine supplementation in mice and older adults. In murine preclinical setup, we tested for safety of various dosing strategies using a sub-chronic, oral administration scenario. Post mortem examination of mice included macroscopic inspection of organs, organ weighing and neoplastic examination after 28 days of supplementation at various concentrations. In addition, animal behavior (i.e., social interaction with humans and cage-mates using standardized score-sheets, food and water intake) and animal bodyweight were controlled during the treatment to detect any negative effects. In the human cohort, a randomized, placebo-controlled, double-blind Phase II study examined safety and tolerability of polyamine supplementation over 3 months. Older adults with subjective cognitive decline (SCD) were chosen as target group, given their elevated risk for developing dementia [16]. Assessments included vital signs, weight, clinical chemistry and hematological parameters of safety, as well as self-reported health status at the end of intervention. Frequency, duration and severity of AEs were assessed throughout the trial. Tolerability was determined by compliance of capsule intake at the end of intervention.

## RESULTS

### Murine preclinical setup

Evaluation of the safety of sub-chronic oral intake of spermidine-rich plant extract was conducted using a repeated dose 28-day oral toxicity protocol recommended by OECD (Organisation for Economic Co-operation and Development, see Methods for Reference). Ten male and 10 female mice per group were thoroughly examined for abnormalities in their behavior, food and water intake and adverse effects by a post-mortem tissue examination (Table 1). Using standardized, blinded, weekly observations of each animal by trained personnel, no abnormalities in social behavior and interaction with humans and cagemates have been observed. No mortalities have been observed during the course of this study. Post-mortem examination showed no significant tissue degeneration or damage other than few cases of cardiac fibrosis spread across several groups including the control group (Supplementary Table 1).

**Table 1:** Characteristics of murine cohort and results of pathological examination after a 28-day oral spermidine supplementation (post mortem analysis).

**Parameter**	**Control group**		**Spermidine group ** (50 g extract/kg bw)*		**p-value** **(intervention)**	
	*Male*	*Female*	*Male*	*Female*	*Male*	*Female*
*Cohort characteristics*						
**n**	10	10	10	10	-	-
**Age** [weeks]	12	12	12	12	-	-
**Bodyweight** [g]	25.9 ± 1.7	21.0 ± 1.1	26.3 ± 1.2	21.4 ± 1.0	1.00	1.00
**Food intake** [g/day]	2.1 ± 0.2	1.9 ± 0.4	2.0 ± 0.3	1.8 ± 0.2	1.00	1.00
**Water intake** [ml/day]	3.1 ± 0.2	3.0 ± 0.1	3.1 ± 0.1	3.1 ± 0.1	1.00	0.27
*Organ weights*						
**Brain** [mg/g bw]	21.4 ± 0.4	21.3 ± 0.4	20.9 ± 0.6	21.8 ± 0.2	1.00	1.00
**Heart** [mg/g bw]	6.9 ± 0.3	4.9 ± 0.2	6.5 ± 0.2	5.1 ± 0.2	1.00	1.00
**Liver** [mg/g bw]	67.4 ± 1.9	52.3 ± 1.7	67.8 ± 2.8	54.4 ± 1.4	1.00	1.00
**Kidney** [mg/g bw]	20.4 ± 0.8	12.9 ± 0.4	22.7 ± 0.6	14.5 ± 0.4	0.18	0.02
**Spleen** [mg/g bw]	7.1 ± 0.3	5.8 ± 0.2	5.9 ± 0.3	6.3 ± 0.2	0.30	0.31
**Muscle** [mg/g bw]	7.1 ± 0.3	5.6 ± 0.2	7.4 ± 0.3	6.1 ± 0.2	1.00	0.31
*Obduction results*						
**Neoplasia**	0	0	0	0	-	-
**Cardiac fibrosis**	2	4	0	0	-	-
**Other fibrotic tissues**	0	0	0	0	-	-

Data are given as mean ± standard deviation (SD). (n = 10 per group and gender), *equivalent of 60 mg/kg spermidine

Food and water intake were not influenced by the supplementation with the polyamine-rich wheat germ extract (Table 1). While bodyweight of animals of all treatment groups did not differentiate from the control group, relative kidney weight to bodyweight ratio was significantly increased in female mice treated with the extreme overdose of 50 g/kg bodyweight. Relative kidney weight was increased by 12% in this group compared to control animals. At lower concentration, this effect was not observed (Supplementary Figure 1). Weights of other examined organs were unaffected by the treatment at all concentrations.

### Polyamine levels in selected murine tissues

**Figure 1:**
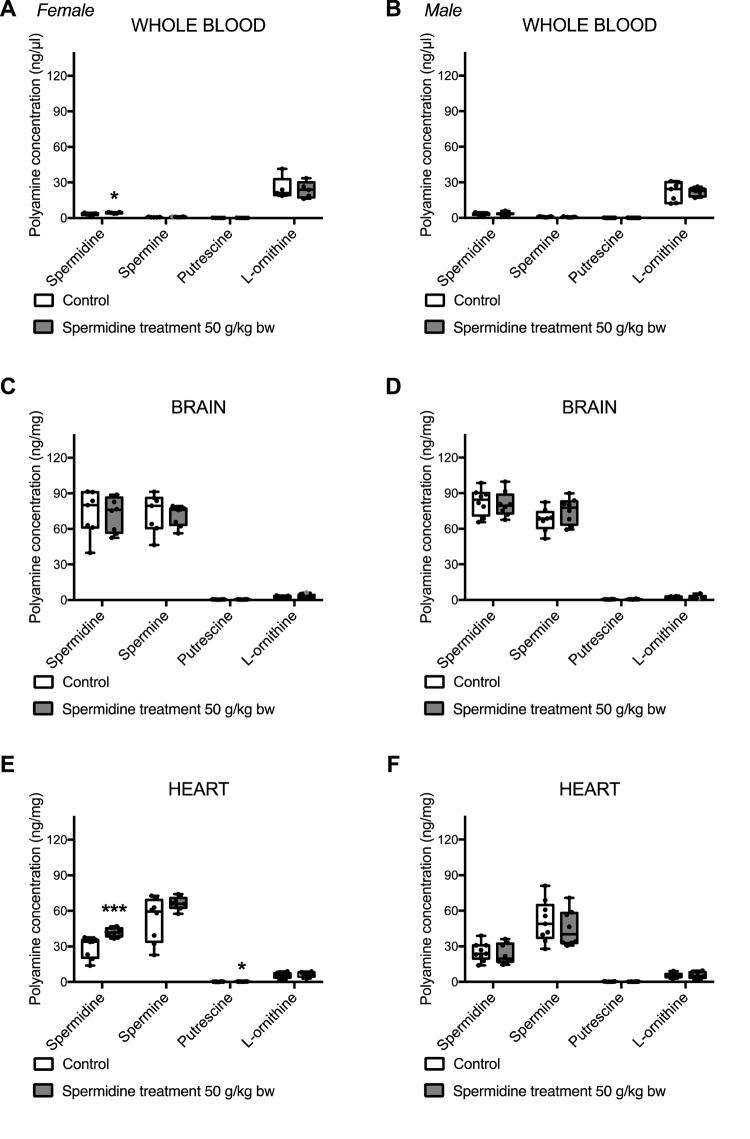
Levels of spermidine, spermine, putrescine, and L-Ornithine in whole blood (**A**,**B**), brain (**C**,**D**) and heart (**E**,**F**) after a 28-day oral spermidine supplementation using spermidine-rich plant extract in female (**A**,**C**,**E**) and male (**B**,**D**,**F**) mice (post mortem analysis). The measurements have been conducted on frozen, murine tissue samples using LC/MS-MS analysis of polyamine metabolites. Data is depicted by box plots extending from 25^th^ to 75^th^ percentile and whiskers ranging from the minimum to the maximum value. Individual data points are marked as dots. (n = 5-10 per gender and group).

Analysis of polyamine levels showed no significant differences in accumulation of polyamines in several tissues after the oral spermidine supplementation in both sexes with few exceptions (Figure 1). While spermidine levels in whole blood of female mice supplemented with highest concentration of spermidine-rich extract in chow was significantly increased compared to the control group after 4 weeks, spermidine blood level in female mice at lower extract concentrations and all male mice remained unaffected by the treatment. Levels of other polyamines in the whole blood remained comparable to the control group in both sexes. Cardiac increase in spermidine and putrescine concentrations after the treatment was observed only in female mice with 50 g/kg bodyweight supplementation, but not at lower concentrations in female or at any supplement concentration in male mice. Concentration of various polyamines in brain tissue showed no correlation to provided treatment. L-Ornithine concentration was not changed in any examined tissues.

### Study enrollment and baseline characteristics of human cohort

**Figure 2:**
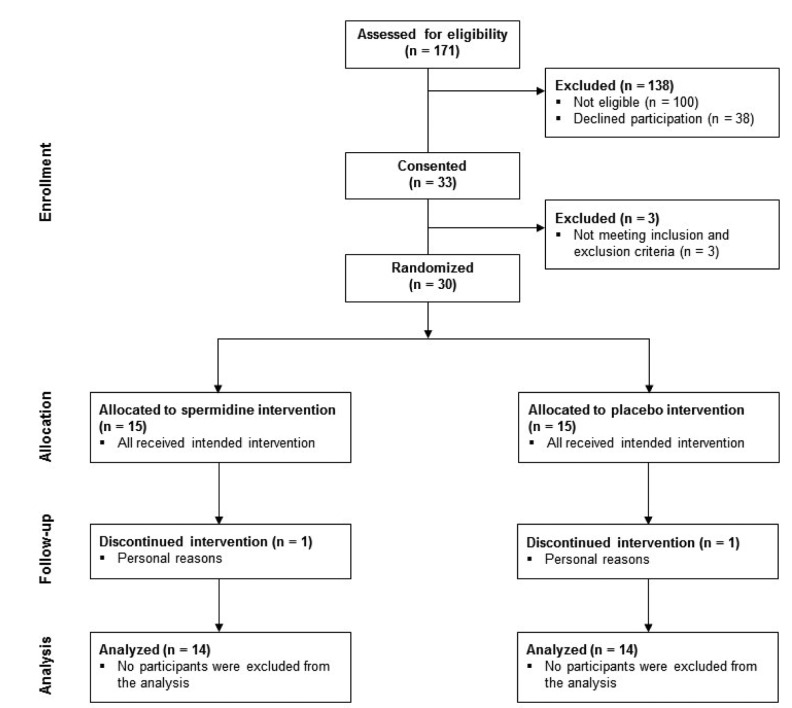
CONSORT diagram indicating the number of participants at each stage of the study

One-hundred-seventy-one adults were interviewed by telephone for study eligibility based on inclusion and exclusion criteria (Figure 2). One-hundred-thirty-eight subjects were excluded, because they were not eligible (n = 100) or declined participation (n = 38). In total, 33 participants were invited from January 2016 to March 2016 for on-site screening. Here, three more individuals had to be excluded because they did not meet inclusion and exclusion criteria. Thirty participants (target sample size of the trial, see clinicaltrials.gov NCT ID: NCT02755246) completed baseline assessment, were subsequently randomized to either spermidine or placebo intervention, and started with the 3-month intervention. During this time, two participants (spermidine group: n = 1; placebo group: n = 1) dropped out due to missing motivation. Twenty-eight participants completed the intervention and were included in the present analysis.

Spermidine (n = 14) and placebo group (n = 14) were comparable for age, years of education, body mass index (BMI) as well as for cognitive and mental status (Table 2). Gender distribution was equal in both intervention groups.

**Table 2:** Baseline characteristics.

**Parameter**	**Placebo group**	**Spermidine group**
**n (women)** [n]	14 (9)	14 (9)
**Age** [years]	69 ± 6 (61-80)	70 ± 5 (60-79)
**BMI** [kg/m^2^]	24.3 ± 2.1 (21-28)	25.3 ± 3.4 (21-33)
**Education** [years]	15 ± 2 (11-18)	16 ± 4 (9-22)
**MMSE** [score]	29.2 ± 0.9 (28-30)	29.1 ± 1.1 (26-30)
**LMS delayed recall** [score]	24.7 ± 5.6 (15-35)	25.0 ± 6.9 (16-35)
**TMT A** [sec]	43.4 ± 14.5 (25-81)	47.5 ± 17.8 (32-90)
**GDS** [score]	2.0 ± 1.5 (0-6)	1.7 ± 1.7 (0-5)

Data are given as mean ± standard deviation (SD) and range for the spermidine and placebo group. BMI: body mass index, MMSE: Mini-Mental State Examination, LMS: Logical Memory Scale, TMT: Trail Making Test, GDS: Geriatric Depression Scale.

Compliance rates, measured by capsule count at follow-up, were on average high in both groups (overall mean compliance rate [Compliance rates, measured SD]: 98.0% Compliance rates, measured 13.0; range 85.7% to 103.1%; spermidine group: 98.8% ± 12.7; range 91.3% to 103.1%; placebo group: 97.2% ± 12.1; range 85.7% to 102.8%).

### Polyamine levels in human whole blood

Blood polyamine (spermidine, putrescine, spermine) and L-Ornithine concentrations did not differ significantly between groups at follow-up (Table 3).

**Table 3:** Levels of spermidine, putrescine, spermine and L-Ornithine before and after 3-month oral spermidine supplementation.

	**Placebo group**		**Spermidine group**			
	Baseline	Follow-up	Baseline	Follow-up	p-value	η2
**Spermidine** [nmol/ml]	8.6 ± 1.8	8.3 ± 1.7	8.5 ± 1.8	8.3 ± 1.9	0.826	0.002
**Putrescine** [nmol/ml]	0.2 ± 0.1	0.2 ± 0.1	0.3 ± 0.1	0.2 ± 0.1	0.493	0.019
**Spermine** [nmol/ml]	4.8 ± 1.1	4.9 ± 1.0	5.6 ± 2.7	5.6 ± 2.1	0.605	0.011
**L-Ornithine** [nmol/ml]	108.7 ± 16.9	102.5 ± 19.2	107.0 ± 19.0	99.5 ± 18.2	0.739	0.005

Data are given as mean ± standard deviation. Differences of parameters at follow-up were estimated via analysis of covariance (ANCOVA). η2: partial eta-squared.

### Weight and vital signs

Weight and vital signs did not differ significantly between groups at the end of intervention (Table 4).

**Table 4:** Weight and vital signs before and after 3-month oral spermidine supplementation.

	**Placebo group**		**Spermidine group**			
	Baseline	Follow-up	Baseline	Follow-up	p-value	η2
**Weight** [kg]	69.5 ± 7.5	69.2 ± 7.0	71.6 ± 10.2	70.6 ± 9.9	0.519	0.017
**SBP** [mmHg]	144.1 ± 15.4	138.5 ± 18.0	144.8 ± 21.3	134.6 ± 15.5	0.378	0.031
**DBP** [mmHg]	87.0 ± 14.0	84.2 ± 10.1	89.2 ± 7.3	83.5 ± 11.2	0.546	0.015
**Heart rate** [bpm]	71.0 ± 12.4	65.2 ± 11.2	68.5 ± 6.5	68.4 ± 8.4	0.131	0.089

Data are given as mean ± standard deviation. Differences of parameters at follow-up were estimated via analysis of covariance (ANCOVA). SBP: systolic blood pressure, DBP: diastolic blood pressure, η2: partial eta-squared.

### Laboratory parameters

Clinical chemistry and hematological parameters did not differ significantly at the end of intervention between the two groups (Table 5).

**Table 5:** Laboratory parameters before and after 3-month oral spermidine supplementation.

	**Placebo group**		**Spermidine group**						
	Baseline	Follow-up	Baseline	Follow-up		p-value		η2	
**Leukocytes** [/nl]	5.9 ± 0.9	6.2 ± 1.5	5.8 ± 1.0		5.8 ± 1.0		0.474		0.021	
**Erythrocytes** [/pg]	4.5 ± 0.5	4.6 ± 0.3	4.6 ± 0.4		4.5 ± 0.4		0.328		0.038	
**Quick** [%]	98.5 ± 11.0	98.0 ± 7.3	101.5 ± 9.3		98.9 ± 6.4		0.914		0.001	
**INR**	1.0 ± 0.1	1.0 ± 0.0	1.0 ± 0.1		1.0 ± 0.0		0.957		0.000	
**Fibrinogen** [g/l]	3.5 ± 0.5	3.6 ± 0.5	3.2 ± 0.6		3.1 ± 0.5		0.060		0.139	
**HbA1c** [%]	5.5 ± 0.5	5.5 ± 0.7	5.4 ± 0.3		5.3 ± 0.3		0.334		0.037	
**IGF** [ng/ml]	102.5 ± 29.0	104.0 ± 20.3	97.4 ± 21.2		101.5 ± 28.3		0.821		0.002	
**Insulin** [mU/l]	7.5 ± 2.3	7.2 ± 3.2	7.9 ± 4.1		7.0 ± 3.5		0.572		0.013	
**Homocysteine** [µmol/l]	12.9 ± 3.3	15.3 ± 4.1	12.4 ± 3.2		12.8 ± 2.6		0.060		0.134	
**Cholesterol** [mg/dl]	225.6 ± 32.4	218.8 ± 40.3	215.6 ± 28.6		210.6 ± 25.2		0.945		0.000	
**HDL** [mg/dl]	65.6 ± 16.0	68.9 ± 14.5	72.5 ± 12.0		75.5 ± 15.2		0.877		0.001	
**LDL** [mg/dl]	147.6 ± 32.6	141.0 ± 36.8	134.4 ± 23.5		126.1 ± 25.2		0.630		0.009	
**Triglycerides** [mg/dl]	96.4 ± 40.3	101.0 ± 44.2	88.5 ± 39.8		84.6 ± 24.6		0.290		0.045	
**Glucose** [mg/dl]	91.9 ± 13.9	92.9 ± 12.4	89.1 ± 7.0		89.9 ± 7.5		0.732		0.005	
*Liver*										
**AS T** [U/l]	28.7 ± 10.7	29.9 ± 7.1	26.0 ± 3.8		25.8 ± 5.3		0.120		0.094	
**ALT** [U/l]	20.9 ± 8.9	20.9 ± 5.9	19.9 ± 3.8		22.2 ± 7.3		0.357		0.034	
*Kidney*										
**Creatinine** [mg/dl]	0.8 ± 0.2	0.8 ± 0.2	0.8 ± 0.1		0.8 ± 0.2		0.204		0.064	
**eGFR**	84.2 ± 12.4	83.9 ± 10.3	82.6 ± 8.2		79.1 ± 10.3		0.125		0.092	
*Inflammation*										
**CRP** [µg/ml]	1.5 ± 1.8	1.6 ± 1.5	2.3 ± 3.4		1.7 ± 1.9		0.848		0.002	
**TNF-alpha** [pg/ml]	2.5 ± 0.6	2.5 ± 1.0	3.0 ± 0.9		2.8 ± 0.5		0.803		0.003	
**IFN-γ** [pg/ml]	8.1 ± 5.5	5.6 ± 1.9	5.8 ± 3.9		7.1 ± 4.7		0.231		0.057	
**IL-2** [pg/ml]	0.3 ± 0.3	0.3 ± 0.2	0.2 ± 0.1		0.2 ± 0.1		0.314		0.041	
**IL-6** [pg/ml]	0.8 ± 0.7	1.0 ± 1.1	0.6 ± 0.4		0.6 ± 0.4		0.606		0.011	
**GM-CSF** [pg/ml]	0.1 ± 0.1	0.2 ± 0.1	0.2 ± 0.1		0.2 ± 0.1		0.458		0.023	

Data are given as mean ± standard deviation. Differences of parameters at follow-up were estimated via analysis of covariance (ANCOVA). One subject of the spermidine group is missing in each of the following parameters: Quick, INR, Fibrinogen, IGF, IL-6, and GM-CSF. ALT: alanine aminotransferase, eGFR: estimated glomerular filtration rate. CRP: c-reactive protein, IFN-γ: interferon-gamma, IL-2: interleukin-2, IL-6: interleukin-6; GM-CSF: granulocyte-macrophage colony-stimulating factor, INR: international normalized ratio of blood clotting, HbA1c: hemoglobin A1c, IGF: insulin-like growth factor-1; HDL: high-density lipoprotein, LDL: low-density lipoprotein, η2: partial eta-squared.

### Adverse events and serious adverse events

Two participants (7.1%) reported a serious adverse event (SAE) during the intervention phase, classified by system organ classes of "immune system disorders" (spermidine group: n = 1) and "infections and infestations" (placebo group: n = 1). Both SAEs were categorized as "omild intensity without permanent damage". Their relationship to intervention was rated as "unlikely related" (allergic reaction after animal contact, after 2.5 months of treatment) and "not related"" (pneumonia), respectively. No specific actions were taken with regard to these events.

### Self-reported health status

Self-reported mental and physical health did not differ significantly between groups at the end of intervention, indicated by mental (p = 0.765, partial eta-squared = 0.004) and physical (p = 0.672, partial eta-squared =0.007) component summary scores of the Short Form-12 (SF-12) health survey questionnaire.

## DISCUSSION

Spermidine supplementation is known to positively influence various age-related health parameters in model organisms, including memory function [8,17], cardiac and renal function [12] as well as autophagy rates [8], thereby promoting longevity [9]. However, safety and tolerability of spermidine supplementation, as a prerequisite for larger clinical trials, have not been established yet in mice and humans. Here we demonstrate for the first time that spermidine supplementation using a spermidine-rich plant extract was safe and well tolerated in mice and in older adults with SCD.

In the murine model, post mortem examination showed no significant changes in organ macroscopic appearance and neoplastic burden after 28 days of spermidine supplementation at various concentrations. An increase of 12% in relative kidney weight in female mice at the highest supplementation dosage (50 g/kg bodyweight) was observed. However, further pathologically relevant signs (i.e., changes in urine excretion, general and social behavior, food and water intake) remained unchanged. The absence of similar effects at other supplement concentrations and after two weeks of washout in female mice, and in parallel no indication of any effects on kidney health in male mice, spermidine-rich plant extract was generally considered as safe for chronic usage in mice. Due to the lack of any intermediary concentrations, the second highest concentration of 5 g/kg bodyweight was used as a NOAEL (No-observed-adverse-effects-level) in mice.

Findings from the murine model were confirmed by the human cohort. We observed no significant differences in weight, vital signs, clinical chemistry and hematological parameters of safety, as well as in self-reported physical and mental health between spermidine and placebo-treated groups at the end of the 3-month intervention. The absence of any significant treatment effects in creatinine and estimated glomerular filtration rate (eGFR) plasma levels, typical biomarkers of kidney function, indicated renal health in the human cohort during spermidine supplementation. High preanalytic sample quality of the human samples, as assured and documented via NeuroHub biomarker management platform and LabVantage 7.0 software, underlined the validity of laboratory results. Moreover, careful monitoring of possible AEs or SAEs during spermidine intervention in humans revealed no evidence for increased (S)AEs in the target group. Only one SAE was observed in each intervention group, both unlikely or not related to the intervention and well treatable. In sum, findings from both murine models and the human cohort argue for excellent safety and tolerability of spermidine supplementation provided as a natural, spermidine-rich plant extract.

Spermidine supplementation using the spermidine-rich plant extract did not significantly alter whole-blood polyamine concentrations in humans and mice at most concentrations. The absence of changes of polyamine levels in the blood may be due to the fast absorption/metabolism rate of polyamines from the intestinal lumen into solid tissues, as observed in an ex vivo rat model by Uda and colleagues [18]. A slight significant increase in whole-blood spermidine levels was observed in female mice fed with the highest spermidine concentration. In this treatment group, spermidine concentration in the heart was also significantly increased, supporting already described uptake of this polyamine by solid tissues in the body. The remaining polyamines and L-Ornithine concentrations in whole-blood samples of both male and female mice as well as humans were unaffected by intervention. Our results reflect the inconsistency of previous studies investigating the effect of polyamine supplementation on endogenous blood polyamine levels. For example, Brodal and colleagues [19] provided evidence that 20-day polyamine supplementation did not alter blood polyamine concentration in rats. Conversely, a 2-month intervention with natto, a polyamine-rich fermented soybean product, led to an increase of spermine, but not spermidine concentration, in blood samples of mice and healthy male participants [7].

Given the growing number of people suffering from dementia, the detection of safe and feasible prevention strategies is of paramount importance [20]. Recent studies have shown that spermidine supplementation has various positive effects on health in aging model organisms, including promotion of autophagy rates and preservation of memory function [8-10]. Thus, spermidine supplementation is suggested as a feasible prevention strategy against age-related health decline, including loss of cognitive function and development of dementia. Given that this study confirmed safety and tolerability of spermidine supplementation in older adults with an elevated risk to develop dementia, the plant extract can be evaluated in larger clinical trials of longer duration.

Strengths and limitations. The main strength of our study is the translational approach to examine safety and tolerability of spermidine supplementation in a murine model and in humans. This enabled us to investigate safety of a polyamine-rich wheat-germ extract directly in various organs and at various (high) concentrations in the murine model, which is not feasible in humans. A possible limitation of our study is the small sample size in the human cohort in both intervention groups and short treatment duration of 3 months in humans and 28 days in mice. However, given the nature of Phase II trials in humans, the target sample of 30 individuals is well within the range of similar safety trials [21].

In conclusion, this study showed that the applied spermidine-rich plant extract was safe and well tolerated in mice and older adults with SCD. These findings open the possibility to investigate the impact of spermidine supplementation on functional and structural brain health as well as on cognition in larger studies with longer intervention times in older adults at risk for dementia.

## METHODS

### Preclinical trial setup and animal housing

In order to examine the safety of the used extracts, a standardized OECD repeated dose oral toxicity study was conducted. [SOURCE: OECD: Guidelines for the testing of Chemicals - "Test 407: Repeated Dose 28-day Oral Toxicity Study in Rodents"] In brief, groups of 10 male and 10 female BALB/cAnNRj mice (Janvier Labs S.A.S.) were provided with either control chow or chow supplemented with a polyamine-rich extract corres-ponding to 0.5 g/kg, 5 g/kg or 50 g/kg animal bodyweight daily intake over the period of 28 days. An additional group of 5 male and 5 female mice was fed with control chow diet for additional 14 days (two weeks washout) after receiving the highest dosage of supplemented diet for 28 days. All experiments were started at the age of 8 weeks.

Animals were housed in a 12h light/dark cycle in type 3 IVC cages (Tecniplast, Model 1284 L) in groups of 5 individuals. Autoclaved nest material and paper houses served as cage enrichment, while access to food and water was provided *ad libitum*. To ensure that the food and water intake were comparable between all groups, drinking water and chow were weighed twice a week. Animals were examined weekly for their external appearance (i.e. examination of the fur, skin and tumors), interaction with cagemates and humans, behavior (i.e. aggressiveness, social interaction, fear, pain, lethargy based on standardized score sheets), and appearance of the fecal and urine excretions. Bodyweight of all animals was recorded by weekly weighing. Animal monitoring was conducted in a blinded manner by an experienced professional.

Pellets for both control and polyamine-enriched chow were prepared using a commercially available base formula (Ssniff, Product-Nr. DP110). All animal experiments were performed in accordance with national and European ethical regulation (Directive 2010/63/EU) and approved by the responsible government agencies (BMWFW-66.007/0012-WF/V/3b/2015).

### Post-mortem animal examination and tissue sampling

At the end of the 28-day long treatment, animals were anesthetized by inhalation of 3-4% isoflurane (ForaneA(r), Baxter Healthcare Corporation) and euthanized by cervical dislocation. Whole blood was collected immediately after euthanasia by cardiac puncture in Ethylenediaminetetraacetic acid (EDTA) coated tubes and stored at -80 °C for polyamine analysis. Blood plasma was prepared from collected EDTA blood by centrifugation at 2000 xg at 4 °C for 15 min and stored at -80 °C for further investigation. After blood collection, animals were thoroughly examined for neo-plasias and visible abnormalities. Further, various organs were weighed, snap frozen in liquid nitrogen and stored at -80 °C for further analysis. The post-mortem examination, sample collection and analysis were conducted in randomized and blinded manner.

### Measurement of polyamine levels in biological samples

A quantitative HPLC-MS/MS-based determination of polyamines in various tissues was performed using whole tissue lysates, as described previously [22]. In brief, polyamines were extracted on ice by incubating samples for 60 min in 5% trichloracetic acid solution. After centrifugation at 25,000 xg at 4 °C for 10 min, supernatant was neutralized with ammonium formiate and stored at -80 °C for subsequent quantification. Prior to the analysis, neutralized samples were derivatized using isobutyl chloroformate [23] and measured using standardized HPLC-MS/MS protocol [22].

### Human study design

A randomized, placebo-controlled, double-blind Phase II study was carried out at the NeuroCure Clinical Research Center, Charité University Hospital. Older adults with SCD and worries (see below for details) were recruited from the memory clinic of the Department of Neurology (Charité University Hospital), a neurology specialist practice (J.B.), and the general population through advertisements. Individuals who were interested in study participation underwent a telephone interview to assess their eligibility. After successful completion of the telephone interview, individuals were invited for on-site screening to the Charité University Hospital. Here, individuals consented to the study and were then screened for inclusion and exclusion criteria based on neuro-psychological tests and questionnaires (see below for further details). If suitable, participants subsequently started baseline assessment. Each participant underwent a standardized medical examination encompassing fasting blood sampling and assessment of weight, height, blood pressure, and pulse. An extensive neuropsychological test and questionnaire battery was administered. Afterwards, the participants were randomly assigned to the two intervention groups (spermidine group and placebo group), using a blockwise (block size of 6) randomization sequence computerized by http://www.randomization.com/, stratified by age. Sequence generation and group allocation was conducted by an investigator with no clinical involvement in the trial. All participants and clinical investigators involved in study implementation were kept blind to the intervention allocation. After the 3-month intervention all baseline assessments were repeated (follow-up). Determination of the sample size (n = 30) was based on the study of Soda and colleagues [7]. The primary outcome of the human trial was cognitive performance and will be published elsewhere. Secondary outcomes of this trial included safety and tolerability measures as reported here.

Participants signed informed written consent prior to on-site screening and received a small compensation for study participation. The study was approved by the Ethics Committee of the Charité University Hospital Berlin, Germany (EA1/233/15), and was carried out in accordance with the declaration of Helsinki. Registration of the study was done in the public registry ClinicalTrials.gov (NCT ID: NCT02755246).

### Participants

Enrolled participants were between 60 - 80 years of age, fluent German speakers, with the presence of SCD in accordance with existing guidelines [16,24]. In detail, all participants had to express subjective cognitive complaints for at least 6 months and related worries, report no deficits in activities of daily living, and score ≤10 in the Geriatric Depression Scale [25]. Eligibility criteria for normal cognitive performance included a Mini-Mental State Examination score ≥26 [26], and performance within -1.5 standard deviation (SD) of age adjusted norms in the Logical Memory II subscale of the Wechsler Memory Scale - Revised [27] and the Trail Making Test A [28,29].

Exclusion criteria encompassed major neurological, internal or psychiatric diseases, malignancies (current or on medical history), untreated thyroid dysfunction or untreated diabetes mellitus, anticoagulation therapy, platelet aggregation inhibitor, known allergy to wheat germs or gluten, histamine intolerance, drug abuse or alcohol dependency, disorders that impair attention, or intake of polyamine supplements before starting the trial.

### Preparation of capsules and dosing

Based on the preclinical data, maximum safe dosage in humans was calculated using the NOAEL of 5 g/kg bodyweight from the murine model, mouse-to-human interspecies factor of 12.3 and factor 10 as safety distance between sub-chronic and chronic application [30]. As a result, doses of up to 41 mg/kg bodyweight or 2.8 g extract containing 3.4 mg spermidine for the average person weighing 70 kg were set as the expected upper safety limit for the treatment.

In total, 750 mg of polyamine-rich plant extract (1.2 mg spermidine, 0.6 mg spermine, 0.2 mg putrescine) and 510 mg cellulose were divided into three Type 00 capsules. This accounts for to an increase of approximately 10-20% of average spermidine intake in developed countries [31,32]. Three placebo capsules per day were filled in sum with 750 mg potato starch and 510 mg cellulose. All capsules were identical in shape, color, taste and smell. Both placebo and spermidine capsules have been provided by TLL The Longevity Labs GmbH (Graz, Austria). Participants of both intervention groups were instructed to follow a regular intake of three capsules a day, one capsule at each main meal (breakfast, lunch, dinner), and not to change their dietary habits during the intervention time. Participants were asked to return capsule bottles at follow-up assessment and the number of remaining capsules was recorded as a measure of compliance and tolerability.

### Examination of physical condition

Physical condition of the participants was evaluated during the standardized medical examination at baseline and follow-up assessment. Following parameters were recorded for each participant at both time points after fasting overnight: weight, height, vital signs as well as any detectable abnormalities of physical condition. Vital signs, including systolic blood pressure, diastolic blood pressure, and heart rate, were assessed in sitting position. Moreover, the participant's weight (in kilograms) was divided by the squared height (in meters) to calculate the respective BMI value. In addition, peripheral blood draw was conducted in sitting or lying position for the analysis of various laboratory parameters.

### Preanalytical preparation and analyses of human blood samples

Peripheral blood samples were obtained between 10am and 12am at baseline and follow-up visit after fasting overnight. Various blood parameters, including liver and kidney values, were analyzed by Labor Berlin (Berlin, Germany) according to standardized procedures. For the analysis of human biomarkers, polyamine levels (spermine, spermidine, putrescine), and L-Ornithine, preprocessing and intermediate storage of these blood samples were performed at the Charité University Hospital, NeuroCure Clinical Research Center. Set-up of standardized workflows, documentation and monitoring of preanalytics, processing, and storage was ensured via NeuroHub biomarker management platform and LabVantage 7.0 software [Märschenz et al. (2017). Implementation of ""NeuroHub" pre-analytics and biomarker management platform for clinical studies allows efficient monitoring of pre-analytical sample quality. Poster presented at: Global Biobank Week *Towards Harmony in Biobanking*, 13-15 September (P-249). Stockholm, Sweden: BBMRI-ERIC, ESBB, ISBER]. For the analysis of human biomarkers EDTA, whole blood was centrifuged at 4 °C and 2000 xg for 15 minutes with brake before plasma was carefully transferred. Plasma and EDTA whole blood had to be stored at -80 °C within one hour after collection. Frozen samples collected at baseline and follow-up were shipped to the Institute of Molecular Biosciences, University of Graz, Austria, for further processing and analyses. Whole blood polyamine levels and L-Ornithine levels were analyzed as described above in the murine model. Common biomarkers of disease and inflammation in humans were measured in EDTA plasma using commercially available V-PLEX human biomarker 40-plex kit (Meso Scale Diagnostics, USA). Samples were analyzed as technical duplicates using the automated QuickPlex SQ 120 (Meso Scale Diagnostics, USA) and validated protocols provided by the manufacturer under consideration of GLP (general laboratory practice) guidelines.

### Adverse events monitoring

During intervention phase, AEs and SAEs were continuously monitored. Participants were instructed to immediately contact the study team in case of any health change or unplanned medical visit. An AE was defined as any untoward medical occurrence associated with the use of a drug in humans, whether or not considered drug related. A SAE was defined as any event that resulted in death or was life-threatening, required hospitalization or prolonged a hospital stay, or resulted in persistent or substantial disability. Both AEs and SAEs were grouped into system organ classes using the Medical Dictionary for Regulatory Activities (MedDRA system, version 13.0, http://www.meddra. org) and summarized including the following parameters: incidence of (S)AEs, intensity (mild, moderate, and severe), and relationship to intervention (not related, unlikely related, possibly related, and related). The occurrence of more than three SAEs was defined as a stopping rule for the trial.

### Self-reported health status

The SF-12 Health Survey [33], a 12 items questionnaire, was administered at baseline and follow-up visit. The SF-12 assesses self-reported physical and mental health and yields two summary scores: mental component summary and physical component summary. Summary scores were computed using published inverting and weighting schemes of individual items [34]. The SF-12 components were shown to be reliable and valid in general and medical populations [33-35].

### Statistical analysis

Statistical analyses were done with the SPSS 22.0 statistical package (PASW, SPSS; IBM, Armonk, NY, USA) and GraphPad Prism v5.0 software (GraphPad Software Inc., La Jolla, USA). For the statistical analyses of changes in mouse organ weights among control and individual treatment groups, we used post-hoc Bonferroni corrected one-way analysis of variance (ANOVA). The statistical analysis of polyamine levels in various mouse tissues between treated and control mice was conducted using student's t-test. In the human cohort, baseline characteristic of intervention groups (spermidine and placebo) and compliance outcomes (capsule count) were compared descriptively using mean, SD, and range. The effect of intervention group on continuous outcomes was estimated using analysis of covariance (ANCOVA) models. Statistical models included intervention group (spermidine and placebo) as independent variable, each of the whole blood polyamine or safety parameter at follow-up as depen-dent variable, and the corresponding baseline measure as co-variate. The level of significance was set at p <0.05 and the effect size measure partial eta-squared for the factor "ogroup" was reported. Effect sizes were defined as small (0.010 to 0.060), medium (0.060 to 0.140) or large (0.140 to 0.200). Baseline and follow-up parameters were characterized within each group using descriptive measures (mean ± SD). AEs and SAEs were evaluated descriptively.

## Supplementary Material

Supplementary File

Study Protocol
