# Supplementary material for: Safety and tolerability of spermidine supplementation in mice and older adults with subjective cognitive decline
Source: Aging (Albany NY). 2018 Jan 8;10(1):19–33. doi: 10.18632/aging.101354 (PMC5807086; doi:10.18632/aging.101354)
Supplement: Supplementary File [file aging-10-101354-s001.pdf]

SUPPLEMENTARY MATERIAL

Table S1. Number of cardiac fibrosis cases per group and gender at the post-mortem tissue analysis.

	No. of cardiac fibrosis cases			
**Group**	0 g/kg bw	0.5 g/kg bw	5 g/kg bw	50 g/kg bw
Male	2	0	1	0
Female	4	1	0	0

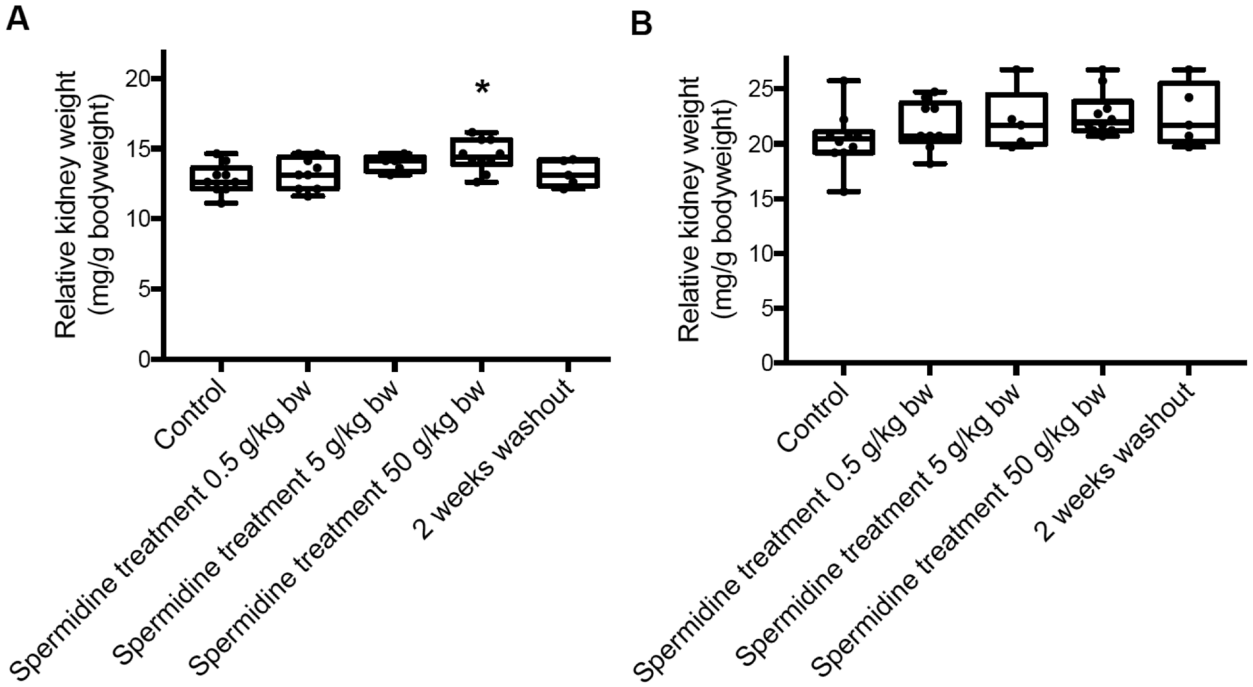

Figure S1. Relative kidney weights of all treatment and control groups after 28-days of oral supplementation in the murine model – In females (**A**) relative kidney weight was significantly increased, but these effects could not be observed at other concentrations or after two weeks washout. Increase in relative kidney weight could not be observed at any concentration in male mice (**B**). Data is presented as mean values with error bars representing the standard deviation, n = 10 (per treatment and sex).
