# Supplementary material for: Safety and tolerability of spermidine supplementation in mice and older adults with subjective cognitive decline
Source: Aging (Albany NY). 2018 Jan 8;10(1):19–33. doi: 10.18632/aging.101354 (PMC5807086; doi:10.18632/aging.101354)
Supplement: Study Protocol [file aging-10-101354-s002.pdf]

1. Titel der Studie	Untersuchung des Einflusses von polyaminreicher Nahrungsergänzung auf die kognitiven Leistungen bei älteren Personen mit subjektiv empfundener Gedächtnisverschlechterung (Smart Age)<br><br>- eine randomisierte, monozentrische, Placebo-kontrollierte, doppelt-verblindete *Proof-of-Concept*-Studie
2. Ethikkommissions -Antragsnummer	(wird von der EK vergeben)
3. Entscheidungen anderer Ethikkommissionen in derselben Sache	In dieser Sache liegen keine Entscheidungen anderer Ethikkommissionen vor (wurden auch nicht beantragt).
4. Gegenstand der Studie und ihre Ziele; Angabe der Hypothesen, getrennt in Haupt- und Sekundärhypothesen sowie der klinischen Parameter (primäre und sekundäre Endpunkte), anhand derer die Hypothesen geprüft werden	**Gegenstand:** Tierexperimentelle Studien haben gezeigt, dass polyaminreiche Nahrung helfen kann, den altersbedingten Erinnerungsverlust bei Fruchtfliegen zu stoppen. Körpereigene Polyamine sind wichtige Produkte des Zellstoffwechsels, deren Konzentration mit dem Alter sowohl bei Fliegen als auch beim Menschen abnimmt. Gleichzeitig nimmt die Anhäufung von verklumpten Proteinen zu, ein Prozess der als wesentlicher Faktor bei Demenzerkrankungen diskutiert wird. Polyamine lösen einen zellulären Reinigungsprozess (Autophagie) aus, der zellulären „Abfall“ (unter anderen Proteinaggregate) bereinigen und somit zum Erhalt kognitiver Gesundheit beitragen kann. Bisher ist ungeklärt, ob die gedächtnisfördernde Wirkung von Polyaminen auch beim Menschen funktioniert.<br><br>Ziel dieser randomisierten, monozentrischen, placebo-kontrollierten, doppelt-verblindeten *Proof-of-Concept* Studie ist, die Sicherheit und Wirkung der nicht-medikamentösen Polyamin-Supplementation bei älteren Menschen mit erhöhtem Demenz-Risiko zu untersuchen. Insbesondere werden Effekte von polyaminreicher Nahrungsergänzung auf kognitive Leistungen (Lern- und Gedächtnisbildung) bestimmt.<br><br>**Primäres Ziel:**<br><br>Zu untersuchen, ob eine gezielte polyaminreiche Nahrungsergänzung (Supplementation) im Vergleich zur Kontrollbedingung (Placebo) die kognitive Leistung verbessert.<br><br>**Primäre Hypothese:**<br><br>Die 3-monatige Intervention (polyaminreiche Nahrungsergänzung) führt im Vergleich zur Kontrollbedingung (Placebo) zu verbesserten kognitiven Leistungen, operationalisiert anhand einer verbalen Lern- und Merkfähigkeitsaufgabe (validierter Gedächtnistest, siehe auch Punkt 10).

	**Sekundäre Ziele:**<br><br>*Periphere Biomarker:*<br><br>Zu untersuchen, ob eine polyaminreiche Nahrungsergänzung im Vergleich zur Kontrollbedingung zu<br>1) einer Erhöhung der Polyamin-Konzentration im Blutplasma führt (u.a. Spermin, Spermidin; gemessen mittels venöser Blutabnahme).<br>2) einer Abnahme von Entzündungsmarkern im Blutplasma (gemessen in venös entnommenen Blut)<br>3) einer Veränderung von vaskulären Prozessen (d.h. der Intima-Media-Dicke und der arteriellen Steifheit)<br>führt.<br><br>**Sekundäre Hypothesen:**<br><br>*Periphere Biomarker:*<br><br>Die polyaminreiche Nahrungsergänzung führt zu<br>1) einer erhöhten Konzentration von Spermin und Spermidin im Blut<br>2) einer Absenkung von Entzündungsmarkern<br>3) einer Verbesserung der vaskulären Prozesse, d.h. der Intima-Media-Dicke der *Arteria carotis interna* (gemessen mittels Dopplersonographie) und der arteriellen Steifheit (gemessen mittels der Pulswellengeschwindigkeit mittels eines Arteriographen)<br><br>**Primärer Endpunkt:** Untersuchung unmittelbar nach Ende der Nahrungsergänzung (3 Monate, T2, Post-Intervention) versus Baseline (T1, Prä-Intervention). Betrachtet wird **die kognitive Leistung.**<br><br>**Sekundäre Endpunkte:** Untersuchung unmittelbar nach Ende der Nahrungsergänzung (3 Monate, T2, Post-Intervention) versus Baseline (T1, Prä-Intervention). Betrachtet werden **die peripheren Biomarker.**
5. Erläuterung der Bedeutung der Studie	In unserer rasant alternden Gesellschaft wird ein 2-4facher Anstieg der Zahl der Demenzerkrankungen erwartet (Bickel, 2000). Daher ist die Suche nach Präventions- und Interventionsstrategien zur Steigerung der Lern- und Gedächtnisbildung von hoher gesundheitspolitischer, wirtschaftlicher und medizinischer Relevanz. Neue Interventions-Strategien sollen pathologische Veränderungen im Gehirn älterer Menschen vorbeugen oder verlangsamen und kognitive Gesundheit fördern.<br><br>Ältere Menschen mit einer subjektiv empfundenen Gedächtnisverschlechterung und diesbezüglichen Sorgen zeigen im Vergleich zu kognitiv intakten Menschen ohne subjektive Beeinträchtigung ein erhöhtes Risiko an Alzheimer-Demenz zu erkranken (Jessen et al., 2014). Bei den Betroffenen ist es daher von besonderem Interesse,

	frühe hirnpathologische Prozesse zu verlangsamen und somit Gedächtnisfunktionen möglichst lange zu erhalten.<br><br>Tierexperimentelle Studien bei Fruchtfliegen haben gezeigt, dass polyaminreiche Nahrung helfen kann, den altersbedingten Erinnerungsverlust zu stoppen. Daher könnte eine polyaminreiche Nahrungsergänzung eine mögliche Maßnahme gegen altersbedingten Gedächtnisverlust darstellen. Bisher sind die direkten Effekte dieser Ernährungsmodifikation auf die kognitiven Leistungen, sowie die zugrundeliegenden neurobiologischen Mechanismen bei kognitiv intakten älteren Menschen mit erhöhtem Demenz-Risiko unbekannt.<br><br>Die geplante Studie wird wertvolle Erkenntnisse über den Einfluss von polyaminreicher Nahrungsergänzung auf die kognitiven Leistungen, sowie die peripheren Blutparameter (insbesondere die köpereigenen Polyamin-Spiegel und Entzündungsparameter) und vaskuläre Prozesse liefern.
6. Welche der folgenden Bestimmungen finden Anwendung<br><br>a) Medizinproduktegesetz<br>-gemäß § 20 MPG (Gerät besitzt nicht die Konformitätserklärung oder diese liegt vor und es wird eine andere Indikation geprüft oder es werden zusätzlich invasive oder andere belastende Untersuchungen durchgeführt) **oder**<br>-gemäß § 23 MPG ?<br><br>b) Strahlenschutzverordnung § 23<br><br>c) Röntgenverordnung § 28 a<br><br>d) Gentechnikgesetz<br><br>e) Datenschutzgesetze	e) Datenschutzgesetze<br>Die Erhebung und Archivierung der Daten folgt dem Berliner Datenschutzgesetz - BlnDSG.
7. Ggf.: Bezeichnung und Charakterisierung der Prüfprodukte<br>(z.B. Geräte bei MPG-Studien; bitte Anlagen beifügen)	Entfällt
8. wesentliche Ergebnisse der vorklinischen Tests oder Gründe für die Nichtdurchführung derselben	**Relevante Untersuchungen zu Polyaminen; allgemein**<br><br>Körpereigene Polyamine (d.h., Putrescin, Spermidin und Spermin) dienen der Aufrechterhaltung von Zellprozessen (Zellwachstum, Zellteilung und Zellüberleben (Minois et al., 2011)), der Regulation von Neurotransmitter-Prozessen und der Modulation von Lern- und Gedächtnisprozessen. Beispielsweise zeigte eine Studie an Mäusen, dass die hippokampale Polyamin-Konzentration (Spermidin, Spermin) mit der Gedächtnisbildung korreliert (Tiboldi et al., 2012).<br><br>**Relevante Untersuchungen zu Polyaminen; Gedächtnis und Altern im Tiermodell**

	In vielen Organismen sinkt die intrazelluläre Polyamin-Konzentration mit zunehmendem Alter in einer Reihe von Organen, so auch im Gehirn (Minois et al., 2011). Bei Fruchtfliegen wurde gezeigt, dass die altersbedingte Reduktion der Polyamin-Konzentration (v.a., Spermidin, Putrescin) im Gehirn mit verringerten Gedächtnisleistungen einhergeht. Externe Polyaminzufuhr (z.B. Spermindin) kann die körpereigene Polyamin-Konzentration erhöhen (Soda et al., 2009b; Gupta et al., 2013), die Lebensspanne bei Hefen, Fruchtfliegen sowie Würmern verlängern und induziert Autophagie sowohl in den genannten Modell-Organismen (Eisenberg et al., 2009) als auch in Mäusen (Brown et al., 2012). Polyaminreiche Nahrung kann zudem den altersbedingten Erinnerungsverlust bei Fruchtfliegen stoppen, was gleichzeitig mit der Zunahme von Autophagie-Prozessen korrelierte (Gupta et al., 2013). Dieser zelluläre Selbstreinigungsprozess kann zellulären „Schrott“, so auch Proteinaggregate, z.B. des Tau Proteins (Schaeffer et al., 2012), im Maus-Modell reduzieren und somit helfen neurodegenerativen Prozessen vorzubeugen. Eine andere Studie von Soda und Kollegen konnte zeigen, dass Mäuse, welche mit polyaminreichem Futter gefüttert wurden, länger lebten und weniger altersbedingte Pathologien auswiesen (Soda et al., 2009a).<br><br>**Untersuchungen zu Polyaminen; Krankheitsprozesse und neuronale Erregung**<br><br>In Säugetieren sind Polyamine in viele Wachstums- und Differenzierungsprozesse von Geweben sowie in Umbauprozesse involviert (Gerner and Meyskens, 2004). Polyamine sind jedoch auch Gegenstand der Krebsforschung, da ein Zusammenhang zwischen gesteigerter Polyaminsynthese, Zellwachstum und Krebs gezeigt wurde (Russell and Snyder, 1968). Eine Zunahme von onkogenen Veränderungen durch polyaminreiche Supplementation konnte jedoch weder bei gesunden Mäusen noch bei Menschen beobachtet werden (Soda et al., 2009b). Es wird vermutet, dass die Erhöhung von Putrescin-Konzentrationen in der Zelle zu einer verstärkten Neoplasie führt (Pegg and Feith, 2007; Welsh et al., 2012). Eine andere Studie an Mäusen hat gezeigt, dass Spermin eine wichtige Rolle bei der Inhibition von pathologischen Veränderungen (so auch Tumorgenesis) spielt, während es das Wachstum von vorhanden Tumoren beschleunigen kann (Soda et al., 2013).<br><br>Polyamine beeinflussen zudem die neuronale Erregbarkeit im Gehirn durch Modulation von Ionenkanälen und Rezeptoren (Ficker et al., 1994), z.B. Glutamatrezeptoren vom Typ NMDA (Ransom and Stec, 1988) sowie einwärts gleichrichtende Kalium-Kanäle (Kir-Kanäle) (Oliver et al., 2000). Polyamine modulieren somit die Erregungsbildung der Neuronen. Damit einhergehend wird eine Dysregulation von Polyamine-Prozessen mit einer Reihe von Erkrankungen in Verbindung gebracht (Park and Igarashi, 2013), wie z.B.

	Epilepsie (Bell et al., 2011), psychische Störungen (Chen et al., 2010), Schlaganfall (Ferchmin et al., 2000) und Diabetes (Kramer et al., 2008; Tersey et al., 2014).<br><br>Die Wirkung von Polyaminen bei diesen Prozessen ist äußerst komplex und kann sowohl neuroprotektive als auch neurotoxische Mechanismen beinhalten. In einer früheren Studie mit langfristiger Zufuhr von polyaminreicher Diät konnten keine entsprechenden Nebenwirkungen bei gesunden Mäusen oder Menschen beobachtet werden (Soda et al., 2009b). Es wird zudem vermutet, dass die altersbedingte Fehlregulation von Polyamin-Konzentrationen zum Teil hinter den verschiedenen Pathologien und Morbiditäten steht, die sich im Alter etablieren können. Eine Studie von Pucciarelli und Kollegen (Pucciarelli et al., 2012) hat gezeigt, dass die Spermidin- und Spermin-Konzentrationen im Verhältnis zur totalen Polyamin-Konzentration im Blut von gesunden neunzig- bis hundertjährigen Menschen erhöht sind. Deshalb vermutet man, dass die Aufrechterhaltung von diesen zwei Polyaminen und die Abnahme der systemischen Putrescin-Konzentration im Blut für ein gesundes Altern von Bedeutung sein könnten.
9. Wesentlicher Inhalt und Ergebnisse der vorangegangenen Studien/Anwendungen der in der Studie zu prüfenden Produkte	**Relevante Untersuchungen zu Polyaminen**<br><br>Beim Menschen wurde eine altersbedingte Reduktion der körpereigenen Polyamin-Konzentration in den Basalganglien (Vivo et al., 2001) und in kortikalen Regionen (Morrison et al., 1995) in post-mortem Studien beschrieben. Durch eine zwei-monatige polyaminreiche Ernährung mit dem fermentierten Sojabohnen-Produktes „Natto“ (durchschnittlich 66 g/Tag, maximal 100 g/Tag) gelang es Soda und Kollegen die körpereigene Polyamin-Konzentration beim Menschen mit einem Faktor von 1,39 (insbesondere Spermin) im Blutplasma zu erhöhen (Soda et al., 2009b). In der gleichen Studie konnte zudem eine Erhöhung der körpereignen Polyamin-Spiegel nach 26-wöchiger polyaminreicher Diät bei Mäusen nachgewiesen werden.<br><br>Man geht davon aus, dass Polyamine Substanzen sind, welche die positiven Wirkungen von Fasten/Kalorienbeschränkung imitieren kann, ohne eine Diät einhalten zu müssen.<br><br>**Polyamin-Konzentration in der Nahrung**<br><br>Basierend auf existierende Studien enthält Natto ca. 50-120 mg/kg Polyamine bzw. 80-100 mg/kg Spermidin und Spermin. Beispielsweise geben Okamoto und Kollegen 116 mg/kg der Polyamine Spermidin und Spermin in Natto an (Okamoto et al., 1997). Eine andere Studie von Zhang und Kollegen (2007, online Referenz: http://en.cnki.com.cn/Article_en/CJFDTOTAL-SHNX200701001.htm) weist Spermidin, Spermin und Putrescin im handelsüblichen Natto nach. Die Autoren geben die Gesamtkonzentration der biogenen Amine in zwei Arten

	von Natto mit 80-100 mg/kg an. Die Ergebnisse von Zhang und Kollegen zeigen, dass 95% der biogenen Amine in Natto Polyamine sind, welche für Menschen nicht akut toxisch sind, unter ihnen 70% Spermidin. Eine dritte Studie von Ibe und Kollegen (Ibe et al., 2013) misst eine Spermidin-Konzentration von 53 mg/kg. Leichte Abweichungen in den Angaben zum Polyamingehalt können darauf beruhen, dass es sich bei Natto um ein Fermentationsprodukt handelt. Die Zusammensetzung des Endprodukts ist u.a. davon abhängig, welche Bakterienstämme verwendet wurden, unter welchen Bedingungen die Fermentation stattgefunden hat und welches Ausgangsmaterial verwendet wurde.<br><br>Die Polyamine, wie Spermin und Spermidin, kommen in nahezu allen pflanzlichen und tierischen Lebensmitteln vor, da sie wichtige physiologische Funktionen besitzen. Es gibt jedoch nur begrenzte Kenntnisse über die Aufnahme von Polyaminen aus den wichtigsten Nahrungsquellen. Einige Studien haben den Inhalt der Polyamine in Lebensmitteln analysiert (Okamoto et al., 1997; Zoumas-Morse et al., 2007) oder die mittlere Aufnahme von Polyaminen in einzelnen Ländern wie Japan (200 µmol, hauptsächlich Putrescin, Nishibori et al., 2007) und Großbritannien (350-500 µmol, Bardocz et al., 1995) geschätzt. Laut Schätzungen in verschiedenen Ländern, darunter Großbritannien, Italien, Spanien, Finnland, Schweden und den Niederlanden (Ralph et al., 1999), beträgt die mittlere Polyamin-Aufnahme für Erwachsene: ca. 212 µmol/Tag Putrescin, 87 µmol/Tag Spermidin und 55 µmol/Tag Spermin. Die durchschnittliche Polyamin-Aufnahme durch die Nahrung liegt damit unterhalb der Richtlinien der *Swedish nutrition recommendations objectified* (SNO), welche eine durchschnittliche Polyamine-Einnahme von 541 µmol /Tag empfehlen (Ali et al., 2011).<br><br>In der oben aufgeführten Studie von Soda und Kollegen (2009b) wurden durchschnittlich 66.4 ± 3.7 g/Tag Natto zugeführt. Das entspricht einer täglichen Polyamin-Zufuhr von ca. 54 µmol/Tag (Angaben der Autoren) und beträgt daher ca. ein Zehntel der SNO Richtlinien.<br><br>**Extraktion von Polyaminen aus Weizenkeimlingen**<br><br>Der Kooperationspartner dieser Studie (Prof. Dr. Frank Madeo, Institut für Molekulare Biowissenschaften, Graz, Österreich) hat eine Methode der Polyamin-Extraktion entwickelt, welches die Herstellung von lebensmitteltauglichen polyaminreichen Pflanzenextrakten für die Produktion gesundheitsfördernder Nahrungsergänzungsmittel ermöglicht. Diese Extraktionsmethode schafft es, ohne starke Säuren, organische Lösungsmittel und/oder Anwendung von gesundheits- oder umweltschädlichen Chemikalien Polyamine aus Pflanzen zu extrahieren. Die Geräte und Techniken, die dabei verwendet werden, wurden schon zur Herstellung von Lebensmitteln und/oder verschiedener Pharmaka verwendet. Somit ist ihre Applikation in der

	Herstellung der an Tieren und Menschen verwendeten Nahrungsmittel unbedenklich und sicher. Als Ausgangsmaterial für die Extraktion werden Weizenkeime verwendet, da sie eine sehr hohe Konzentration an Polyaminen (v.a. Spermidin) enthalten und seit Jahrhunderten in der menschlichen Ernährung verwendet werden. Die Effizienz der Extraktion wurde im Labor unter der Leitung von Prof. Dr. Frank Madeo weiter optimiert.<br><br>In dem zu verabreichendem Extrakt sind vor allem Spermidin und Spermin angereichert (mit 13,3 mg Spermidin und 6,34 mg Spermin pro 1 g Extrakt). Ferner enthält 1 g Extrakt: 2,38 mg Putrescin, 0,060 mg Cadaverin und 1,95 mg L-Ornithin. In der Kombination mit einer normalen Ernährung, liegt die Einnahme der geplanten Tagesdosis von 750 mg Extrakt (sogar im Fall einer mehrfachen Überdosierung) unter dem errechneten NOAEL (*No-Observed-Adverse-Effects Level*) für Menschen von 29 mg/kg Köpergewicht (KG)/Tag für Cadaverin und Putrescin (Til et al., 1997). Auch im Fall von Spermidin und Spermin ist das der Fall, bei denen das NOAEL bei 13,5 mg/kg KG/Tag, bzw. bei 3,1 mg/kg KG/Tag liegt (Til et al., 1997). Das NOAEL für L-Ornithin liegt bei Menschen über 500 mg/kg KG/Tag (Ishida et al., 2013) und bleibt somit unerreichbar bei der geplanten Verabreichung des Extraktes.<br><br>Die Verabreichung von verkapselten Weizenkeimextrakten wird täglich in Form von zwei Kapseln von jeweils 375 mg Extrakt pro Kapsel erfolgen und führt somit zu einer Tagesdosierung von 750 mg Extrakt bzw. 10 mg Spermidin, 4,8 mg Spermin, 1,8 mg Putrescin, 0,045 mg Cadaverin und 1,49 mg L-Ornithin pro Tag. Diese Dosierung ist vergleichbar mit der verabreichten Menge an Polyaminen in der früheren Substitutionsstudie aus Japan (Soda et al., 2009b). Die Studie von Soda und Kollegen hat bei Menschen ca. 10 mg/Tag Spermidin (Maximalwert) eingesetzt. Diese Menge an Polyaminen wäre auch denkbar bei einer normalen und gezielten Ernährung (z.B. 200 g gekochten Sojabohnen, Ali et al., 2011) und sollte dementsprechend zu keinen Nebenwirkungen führen.<br><br>Um die Sicherheit der Extrakte für die Anwendung an Tieren und Menschen zu überprüfen, wurde eine chemische Lebensmittelanalytik an den Extrakten durchgeführt. Ausgenommen von Didecyldimethylammoniumchloride (DDAC) und Benzalkoniumchlorid (BAC) wurden keine Spuren von Toxinen und Pestiziden in den Extrakten nachgewiesen. Die gemessene Konzentration betrug 2,7 mg/kg Extrakt für DDAC und 1,2 mg/kg Extrakt für BAC (Summe). Bei einer Dosierung von 750 mg Extrakt pro Tag, wird die von der EFSA (*European Food Safety Authority*) vorgegebene maximale Grenze von diesen beiden

	Subtanzen nicht erreicht. Die Tagesdosierung für das DDAC 0,029 µg/kg KG* und für das BAC (Summe) 0,013 µg/kg KG[*] bei einer Einnahme von 750 mg Extrakt[†] betragen. Somit wird bei einem Erwachsenen das ADI (*Acceptable Daily Intake*) von jeweils 100 µg/kg KG/Tag für diese zwei Substanzen nicht überschritten.<br><br>Weitere Inhaltsstoffe, die bei der Untersuchung der Extrakte über der Detektionsgrenze lagen, waren verschiedene B-Vitamine und Cadmium (Cd, 0,15 mg/kg Extrakt). Bei der Dosierung von 750 mg Extrakt pro Tag wird eine wöchentliche Menge von 0,0016 µg Cd/kg KG*/Tag zugeführt und liegt somit weit unter den vorgeschriebenen 0,36 µg Cd/kg KG/Tag[‡]. Im Fall der B-Vitamine ist die gemessene Menge zu gering, um einen Effekt auf die Gesundheit bei der geplanten Dosierung zu haben. Weitere Bestandteile des Extrakts sind pflanzliche Proteine und Zucker, wobei diese keinen Einfluss auf die menschliche Gesundheit haben sollten. Die Extrakte eignen sich allerdings nicht für die Anwendung an Personen mit Gluten-Unverträglichkeit.<br><br>**Verträglichkeitsstudien im Mausmodel**<br><br>Die 28-Tage Verträglichkeitsuntersuchung an Mäusen (durchgeführt nach OECD-Richtlinie für Untersuchungen von chemischen Substanzen “Test 407: Repeated Dose 28-day Oral Toxicity Study in Rodents”) zeigte keine Veränderungen im Fress-, Trink- oder generellen Verhalten durch die Behandlung (qualitative Auswertung). Dabei wurden folgende Mengen am Extrakte täglich über das Futter an Mäuse verabreicht: 0,56 g/kg KG/Tag (niedrig), 5,6 g/kg KG/Tag (mittel) und 56 g/kg KG/Tag (hoch). Diese entsprechen den 10 mg Spermidin/kg KG/Tag, 100 mg/kg KG/Tag, bzw. 1000 mg Spermidin/kg KG/Tag. Die Organ-Gewichte aller untersuchten Organe ausgenommen von Milz und Nieren blieben durch die Behandlung unverändert. Das Nierengewicht (relativ zum KG) wurde bei der hohen Dosierung um 9,9% bei beiden Geschlechter erhöht (*p-value*: 0,005). Aufgrund dieser Messung wird angeraten, Personen mit Nierenerkrankungen von der Studie auszuschließen. Das Milzgewicht (relativ zum KG) wurde nur bei Männchen bei der höchsten Dosierung um 18% gesenkt (*p-value*: 0,004). Bei den Weibchen blieb das Milzgewicht (absolut und relativ zum KG) unverändert. Das Vorkommen

* Berechnung für eine 70 kg schwere Person

† European Food Safety Authority, 2013; Evaluation of monitoring data on residues of didecyldimethylammonium chloride (DDAC) and benzalkonium chloride (BAC). EFSA supporting publication 2013:EN- 483. 30 pp.

‡ Scientific Opinion of the Panel on Contaminants in the Food Chain on a request from the European Commission on cadmium in food. The EFSA Journal (2009) 980, 1-139

	von Herzfibrosen wurde um 80% Prozent durch die Behandlung bei der niedrigen und mittleren Dosierung gesenkt. Bei der höchsten applizierten Konzentration wurden keine Fälle der Herzfibrose identifiziert. Da die am Menschen verwendete Dosierung um Faktor 100 geringer als die im Tierversuch verwendete niedrige Konzentration ist, erwarten wir keine negativen Effekte von den Extrakten an Menschen.<br><br>In der Literatur wird eine Inhibierung von Kir-Känlen durch kationische Eigenschaften der Polyamine (vor allem Spermin) beschrieben (Pegg, 2009). Diese könnte sogar die unterschiedliche Dynamik der Aufnahme der Kalium-Ionen durch die Kir-Kanäle in verschiedenen Geweben erklären (Yan et al., 2005). Trotz vieler *in vitro* Studien zu diesem Thema, wurden nur wenige Tiermodelle entwickelt, um diese Eigenschaft der Polyamine und ihre physiologische Relevanz *in vivo* zu untersuchen (Pegg and Wang, 2009). Vor allem, wurden keine positiven Studienergebnisse zu einer solchen Modulation bei der oralen Einnahme von Polyaminen veröffentlicht (Pegg, 2009). Trotz mangelnder Information können diese Effekte bei einer ausreichend dosierten Supplementation nicht ausgeschlossen werden. Sie könnten sogar die beobachteten positiven Effekte auf die myokardiale Funktion bei der lebenslangen oralen Supplementation erklären (Madeo, unpublizierte Daten).<br><br>Im Rahmen dieser Studie werden Polyamin-Mengen eingesetzt, die bei einer ausgewogenen Ernährung problemlos erreicht und sogar überschritten werden können. Die Tagesdosis von 110 µmol Spermidin und Spermin zusammen pro Person kann nur sehr schwer eine aus der Literatur bekannte Konzentrationserhöhung von 5 µM auf 20 µM Spermin im intrazellulären Raum (Yan et al., 2005) erzielen. Deswegen schätzen wir die Gefahr für die Modulierung der Kir-Kanälen durch Extrakt-Applikation als gering ein, denn sie ist vergleichbar zu einer vegetarischen, Sojaprodukt-reichen Diät.
10. Beschreibung der vorgesehenen Maßnahmen/Untersuchungsmethoden und eventuelle Abweichungen von den in der med. Praxis üblichen Maßnahmen/Untersuchungen (was ist „Routine“, was wird davon abweichend in der Studie gemacht?)	**Studienablauf**<br><br>• **Aufklärung und Einwilligung der Teilnehmer**<br><br>Der Proband wird über eine mögliche Teilnahme an der Studie schriftlich und mündlich aufgeklärt. Wenn nach ausreichender Bedenkzeit das schriftliche Einverständnis zur Studienteilnahme abgegeben worden ist (informed consent), erfolgt die Aufnahme in die Studie, und eine einmalige Identifikationsnummer wird zur späteren Pseudonymisierung erstellt.<br><br>• **Eingangsuntersuchung**<br><br>Hier wird geklärt, ob der Studienteilnehmer an der Studie ohne gesundheitliche Risiken teilnehmen kann. Liegen keine Bedenken vor, kann der Studienteilnehmer entscheiden, ob er an der Studie freiwillig teilnehmen möchte. Die Einwilligung kann jederzeit widerrufen werden. Folgende

Parameter werden diesbezüglich erfasst:

- Alter und weitere demografische Angaben
- MRT-Tauglichkeit
- neuropsychologisches Screening anhand standardisierter neuropsychologischer Tests: Mini Mental State Examination (Folstein et al., 1975), Fragen zur subjektiv empfundenen Gedächtnisverschlechterung und diesbezüglichen Sorgen (Jessen et al., 2014), normale Gedächtnisleistung (Wechsler Memory Scale Logical Memory II (Wechsler, 1997b))
- Geriatric Depression Scale (GDS) (Yesavage et al., 1982)

- **<u>Baseline-Untersuchungen</u>**

Folgenden Baseline-Untersuchungen werden erhoben:

a) Ausführlichere neuropsychologische Testung anhand standardisierter Testverfahren. Folgende Funktionsbereiche werden geprüft: Verbales und visuell-räumliches Gedächtnis, Aufmerksamkeit, Exekutive Funktionen
b) körpereigene Polyamin-Konzentration im Blutplasma
c) periphere Parameter wie Entzündungsmarker, Insulin, Glukose, HbA1c, Neurotrophine, Lipid- und Fettsäureprofil im Blut,
d) Bildgebung von vaskulären Prozessen (Dopplersonographie der *Carotis interna* zur Bestimmung der Intima-Media-Dicke und die Messung der arteriellen Steifheit mittels Arteriographen)
e) ernährungsrelevante Parameter, wie Körpergröße, Gewicht, Taille-Hüft-Verhältnis
f) validierte Fragebögen erhoben, u.a., zur Schlafqualität, zum Lebensstil (Rauch- und Trinkgewohnheiten, zur körperlichen und geistigen Aktivität, zur Lebensqualität, zum allgemeines subjektives Befinden (*Positive and negative Affective Schedule*, PANAS (Watson et al., 1988)
g) Bildgebung des Gehirns (Ausschluss relevanter Begleiterkrankungen, z. B. Tumoren, territoriale Schlaganfälle)

- **<u>Untersuchungen nach Intervention</u>**

Nach 3 Monaten Intervention (T2) werden die oben beschriebenen Messungen a-f durchgeführt.

- **<u>Intervention:</u>**

Die **Versuchsgruppe** erhält eine Polyamin-Supplementation in Kapselform (750mg Weizenkeim-extrakt, äquivalent 28 Weizenkeimen/Tag) Der Polyamin-Gehalt pro Kapsel (375 mg Extrakt, halbe Tagesdosis) beträgt 5 mg Spermidin, 2,4 mg Spermin, 0,9 mg Putrescin und 0,0225 mg Cadaverin. Diese Menge ist äquivalent zu 10 mg/Tag (Spermidin) welches über eine Nahrungsergänzung von max. 100 g

	Natto/Tag in der früheren Studie zugeführt wurde, die körpereigene Polyamin-Konzentration erhöhte und gut (ohne Nebenwirkungen) vertragen wurde (Soda et al., 2009b).<br><br>Die **Kontrollgruppe** erhält Placebo-Kapseln (aus Kartoffelstärke). Alle Kapseln sind identisch von ihrem äußeren Erscheinungsbild (Blindung), und werden für die Probanden in gleicher Weise zu den Mahlzeiten aufgenommen.<br><br>Die Studienteilnehmer erhalten in einer individuellen Eingangssitzung (1 Stunde) detaillierte Information zur Kapseleinnahme, nach 3 Monaten werden sie erneut einbestellt und die übrig gebliebenen Kapseln werden zur Feststellung der Compliance gezählt.<br><br>**Versuchsplan:**<br><br>Es handelt sich um ein randomisiertes, Placebo-kontrolliertes Studiendesign. Die Studienteilnehmer werden in Gruppen randomisiert (stratifizierte Randomisierung nach Alter und Geschlecht). Alle Gruppen nehmen an einer 3-monatigen Intervention oder Placebo-Bedingung teil.<br><br>Während der Interventionszeit sind die Teilnehmer geblindet gegenüber der Versuchsbedingung (Verum, Placebo). Ebenso ist der wissenschaftliche Mitarbeiter, der die Messungen erhebt, vor, während und nach der Intervention bezüglich der Versuchsbedingung geblindet.<br><br>**Verlaufsuntersuchungen:**<br><br>Während der Interventionsphase wird ein Gespräch (Telefonat) eingeplant. Dabei erfolgen die Prüfung der Compliance und ein kurzes Interview zur Intervention.<br><br><br>**Beschreibung der verwendeten Methoden/ Aufgaben**<br><br><u>*Psychometrie:*</u><br><br>Validierte Papier-und-Bleistift sowie computerbasierte Tests werden eingesetzt. Die visuelle und verbale Lern- und Merkfähigkeit der Studieneilnehmer wird u.a. anhand folgender Tests bestimmt: *Auditory Verbal Learning Test* (AVLT) (Lezak, 2004), *People-and-Door Test* (Baddeley et al., 1995), computerbasierte visuelle Gedächtnisaufgaben, sog. *Pattern Separation Task* oder *Recognition Memory test* (Duzel et al., 2011; Stark et al., 2013). Exekutive Funktionen (Aufmerksamkeit und Arbeitsgedächtnis) werden u.a. durch folgende Tests geprüft: *Digit Span* (Härting et al., 2000), *Corsi Block Tapping* (Kessels et al., 2000), *Trail Making Test A and B* (Reitan and Herring, 1985), *Digit Symbol Coding* (Wechsler, 1997a), semantische und phonologische Wortflüssigkeit (Regensburger Wortflüssigkeits-Test, RWT), Stroop Test (Trenerry et al., 1989) sowie Untertests zur Aufmerksamkeitsprüfung (TAP) (Zimmermann and Fimm, 1993). Die psychometrische Testbatterie wird ca. 60 Minuten dauern. Es wird mit der hiesigen Neuropsychologin Frau

	Dr. U. Kopp zusammengearbeitet.<br><br>*Magnetresonanztomographie (MRT) (Baseline) :*<br><br>Die Studienteilnehmer erhalten zur Baseline eine strukturelle MRT-Messung des Gehirns, die mittels eines 3-Tesla-MRT-Scanners (Siemens) im Campus Mitte (BCAN-Scanner) akquiriert wird. Falls bereits ein MRT vorhanden ist und dieses im BCAN innerhalb von 12 Monaten vor der Baseline Untersuchung gemessen wurde, wird dieses im Rahmen der geplanten Untersuchung verwendet.<br><br>Es werden drei strukturelle Sequenzen wie folgt gemessen: eine T1-gewichtete 3D Sequenz mit isotroper Auflösung von 1×1×1 mm (oder 0,5×0,5×0,5 mm), eine *Fluid Attenuated Inversion Recovery* (FLAIR) Sequenz und eine *Difusion tensor imaging* (DTI) Sequenz. Die Messdauer im MRT beträgt ca. 30 Minuten.<br><br>Die MRT-Bilder werden neuroradiologisch bezüglich relevanter Pathologien (v. a. Tumoren, territoriale Hirninfarkte) beurteilt.<br><br>*Laborparameter (Blut):*<br><br>- Polyamin-Konzentration<br>- Basisparameter (kleines Blutbild, Leber- und Nierenwerte, Triglyceride, LDL- und HDL-Cholesterin, Gerinnung (PTT, Quick)) (Kohlmeier, 1995; Yao et al., 2002)<br>- Glucose, Insulin, HbA1c, Leptin<br>- Entzündungsparameter: hsCRP, TNFα, IL-6, IL-1b, Fibrinogen<br><br>Die Analyse der körpereigenen Polyamin-Konzentration wird im Labor von Prof. Dr. Sigrist, Freie Universität Berlin, Institut für Biologie/Genetik, Berlin durchgeführt. Alle übrigen Messungen werden durch das Auftragslabor IMD Berlin durchgeführt.<br><br>*Dopplersonographie:*<br><br>Um Gefäßveränderungen vor und nach der Intervention zu erfassen, wird mittels Sonographie (Ultraschall) die Intima-Media-Dicke (IMD; *intima-media-thickness* – IMT) der *Arteria carotis* beidseitig bestimmt, unterstützt durch ein neuartiges Software-Tool („QIMT“, von der Firma Esaote).<br><br>*Messung der Arteriellen Gefäßsteifigkeit:*<br><br>Zur Messung der Gefäßelastizität wird ein Arteriograph verwendet und es werden zwei Parameter bestimmt: Die Pulswellengeschwindigkeit und der Augmentationsindex. Als Pulswellengeschwindigkeit bezeichnet man die durch die Kontraktion des Herzens erzeugte Druckwelle, die sich mit einer bestimmten Geschwindigkeit (normal 6 - 12 m/s) durch den arteriellen Gefäßbaum bewegt.<br><br>**Repräsentativität Studienteilnehmerauswahl**

	Zur Erfassung aller für die Studie geeigneten Teilnehmer wird vom Prüfarzt eine Liste geführt (Trial-Register), aus der hervorgeht, wie viele die Ein- und Ausschlusskriterien erfüllen, jedoch aus verschiedenen Gründen nicht in die Studie aufgenommen wurden.<br><br>**Vorzeitige Beendigung der Studie für den einzelnen Studienteilnehmer**<br><br>Jeder Studienteilnehmer kann ohne Angabe von Gründen aus der Studie ausscheiden. Mögliche Gründe für einen Abbruch können sein:<br><br>- Widerruf der Einwilligung zur Studienteilnahme<br>- Auftreten von schwerwiegenden Ereignissen, die einen Abbruch notwendig machen
11. Bewertung und Abwägung der vorhersehbaren Risiken und Nachteile der Studienteilnahme gegenüber dem erwarteten Nutzen für die Studienteilnehmer und zukünftig erkrankte Personen (Nutzen-Risiko-Abwägung)	Insgesamt ist das Risiko gesundheitlicher Schäden durch die Untersuchung unter Beachtung der Kontraindikationen als minimal einzuschätzen (Details siehe Punkt 11c).<br><br>Die Studienteilnehmer werden über alle möglichen Risiken der Studie aufgeklärt und darüber, dass sie ihre Zustimmung jederzeit ohne Angabe von Gründen zurückziehen können (s. beigefügte Teilnehmerinformation). Der zu erwartende „Drop-out“ von Probanden ist durch die Probandenzahl (siehe Fallzahlanalyse) ausgeglichen,<br><br>Alle durch die Studie auftretenden negativen Effekte (AE und SAE s.u.) werden standardmäßig dokumentiert.<br><br>***Adverse Events* (AE)** der geplanten Studie: Jedes unerwünschte oder ungünstige Vorkommnis, das einen Studienteilnehmer betritt, einschließlich abnormer Befunde (z.B. abnorme körperliche Untersuchungen oder Laborbefunde), Symptome oder Krankheiten, welche während der Studienteilnahme auftreten, unabhängig davon, ob es mit der Studienteilnahme in einem direktem Zusammenhang steht.<br><br>***Serious Adverse Events* (SAE) der** geplanten Studie: Jedes Vorkommnis welches:<br>- lebensbedrohlich ist<br>- zur Hospitalisierung führt<br>- zu schwerwiegenden körperlichen Beeinträchtigungen führt<br>- jedes weitere Vorkommnis, was vom Studienleiter als erhebliche Gefahr beurteilt wird<br><br>Im Falle des Auftretens von SAE:<br><br>Der Prüfarzt gibt zunächst eine Einschätzung ab, ob ein kausaler Zusammenhang mit der Einnahme des Prüfpräparates für möglich gehalten wird. Da es sich um eine doppelt verblindete Studie handelt, erfolgt dies ohne Kenntnis der Gruppenzugehörigkeit (Intervention vs. Placebo).<br><br>Die Zuordnung zu den einzelnen Behandlungsgruppen erfolgt erst nach der Entblindung im Rahmen der

	Auswertung der Studie.<br><br>Ausnahme ist eine Notfallentblindung, die der Prüfarzt vornehmen darf, wenn die Entscheidung über die Weiterbehandlung des betroffenen Patienten von der Kenntnis der Studienbehandlung abhängig ist. Sollten mehr als drei der eingeschlossenen Studienteilnehmer in der Interventionsgruppe SAEs aufweisen, wird die Studie abgebrochen.
a. Voraussehbarer therapeutischer Nutzen für die Studienteilnehmer (**individueller Nutzen** für den einzelnen Studienteilnehmer)	Der individuelle Nutzen für die Studienteilnehmer mit erhöhtem Demenzrisiko ist nicht sicher vorhersagbar. Aus tierexperimentellen Studien ist bekannt, dass polyaminreiche Nahrungsergänzung positive Effekte, z.B. eine Verbesserung der Gedächtnisleistung und eine Zunahme der Autophagie-Prozesse, erzielen kann. Daher ist nach Einschätzung der bisherigen Datenlage ein positiver Effekt für die Studienteilnehmer zu erwarten. Selbst wenn individuell „nur" eine Verbesserung im Wohlbefinden erzielt werden könnte, wäre das als positiv für den Einzelnen zu bewerten.
b. Voraussehbarer medizinischer Nutzen für zukünftig erkrankte Personen **(Gruppennutzen)**	Bei positivem Effekt der Intervention würden sich neue Möglichkeiten in der Behandlung von kognitiver-intakten älteren Personen mit subjektiv empfundener Gedächtnisverschlechterung ergeben. Bei der polyaminreichen Nahrungsergänzung handelt es sich um eine einfach anwendbare Applikation. Zudem weist der nicht-invasive Behandlungsansatz ein sehr gutes Sicherheitsprofil auf (Soda et al., 2009b).
c. **Risiken** und Belastungen für die Studienteilnehmer (alle im Einzelnen auflisten)	Die Studienteilnehmer werden während allen Stadien der Studie unter ständiger ärztlicher Aufsicht und Behandlung stehen.<br><br><u>Einnahme einer Polyamin-Supplementation in Kapselform, gewonnen aus Weizenkeimlingen:</u> Polyamin-reiche Nahrungsmittel wie Natto (aus Soja) oder Weizenkeimlinge werden im Allgemeinen sehr gut vertragen. Grundsätzlich sind wie bei allen Nahrungsmitteln Unverträglichkeiten oder Allergien auf das betreffende Nahrungsmittel möglich. Daher sollte bei bekannter Unverträglichkeit oder Allergie gegen das betreffende Nahrungsmittel dieses nicht zugeführt werden. Wie bei allen Nahrungsmitteln kann es zu leichten Magen-Darm-Beschwerden kommen, bei Weizenkeimlingen zu Aufstoßen sowie Durchfall, die aber nach Absetzen komplett sistieren. Das Aufstoßen kann durch Einnahme mit den Mahlzeiten verringert oder verhindert werden und wird den Probanden daher empfohlen.<br><br>Da Weizenprodukte grundsätzlich Gluten enthalten, sollte bei bekannter Unverträglichkeit keine Polyamin-Substitution mit Weizenkeimderivaten durchgeführt werden.<br><br>Die Polyamine Putrescin, Spermin und Spermidin in der Nahrung verzögern zudem die Histamin-Oxidation durch die Diaminoxidase (DAO). Daher wird bei bekannter Histaminintoleranz keine Polyamin-Supplementation durchgeführt.

	In vergleichbaren Arbeiten zur Polyamin-Substitution in hohen Dosen wurden keine darüber hinausgehenden spezifischen Nebenwirkungen berichtet (Soda et al., 2009b).<br><br>*Psychometrische Testung:* Ist, abgesehen von Ermüdungserscheinungen im Einzelfall, nicht mit speziellen Risiken verbunden.<br><br>*MRT:* Ist ein nicht-invasives Verfahren, das weltweit angewandt wird und bei Beachtung der relevanten Ausschlusskriterien (z. B. magnetisches Metall im Körper) kein Risiko für die Studienteilnehmer darstellt.<br><br>*Bei venöser Blutentnahme* kann es zu Komplikationen wie Hämatome, Infektion oder heftiger Schmerz an der Einstichstelle kommen. Diese Komplikationen treten jedoch extrem selten bei der Behandlung durch erfahrene Ärzte auf und sind außerdem gut behandelbar und vollständig reversibel.<br><br>Die Messung der *Intima-Media-Dicke (Doppler-Sonographie) und der Gefäßelastizität (Arteriograph)* im Rahmen unserer Untersuchungen ist schmerzlos und nimmt nur wenig Zeit in Anspruch.
12. Maßnahmen zur Risikobeherrschung	Folgende Sicherheitsmaßnahmen sollen bei dieser Studie Risiken zu minimieren helfen (siehe Punkt 11):<br><br>1) Die Einhaltung der Ein- und Ausschlusskriterien wird strengstens überwacht. Die Probanden können die Untersuchung jederzeit abbrechen (auch während des Versuchs).<br><br>2) Alle Teilnehmer erhalten eine neurologische und medizinische Eingangsuntersuchung.<br><br>3) Alle Untersuchungen werden von erfahrenen Untersuchern durchgeführt. Im Falle unerwünschter Nebeneffekte ist Zugang zu Notfallmaßnahmen umgehend gegeben. Um dennoch nicht auszuschließende Risiken zu minimieren, werden alle Versuchsteilnehmer sorgfältig nach möglichen Ausschlusskriterien befragt. Sie werden über alle möglichen Risiken der aufgeklärt und darüber, dass sie ihre Zustimmung jederzeit ohne Angabe von Gründen zurückziehen können.<br><br>4) Die MRT-Untersuchung des Gehirns wird nach genauer Befragung bezüglich möglicher Risiken oder Kontraindikationen (z. B. Herzschrittmacher, intrakranielle Metallclips) durchgeführt. Unter Beachtung dieser Kontraindikationen ist das Risiko der MRT-Untersuchung minimal.<br><br>5) Die Probanden werden aufgefordert, sich bei Auftreten von gesundheitlichen Störungen unverzüglich mit dem Untersucher oder mit der Neurologischen Poliklinik in Verbindung zu setzen. Entsprechende Telefonnummern

	werden ausgehändigt.
13. Abbruchkriterien	Jeder Teilnehmer kann ohne Angabe von Gründen aus der Studie ausscheiden. Mögliche Gründe für einen Abbruch können sein:<br>- Widerruf der Einwilligung<br>- Nicht-Toleranz der Bildgebungs-Untersuchung durch den Studienteilnehmer<br>- Nicht-Toleranz der regelmäßigen Einnahme der Polyamin-Supplementation oder Placebo-Kapseln<br>- Auftreten von anderen schwerwiegenden Ereignissen, die einen Abbruch notwendig machen
14. Anzahl, Alter und Geschlecht der betroffenen Personen	30 kognitiv intakte ältere Personen mit subjektiv empfundener Gedächtnisverschlechterung und diesbezüglichen Sorgen im Alter zwischen 60 und 80, sowohl Männer als auch Frauen (siehe Fallzahlberechnung- nur Pilotstudie mit 15 vs. 15 Probanden, ohne weitere Poweranalyse).
15. Biometrische Planung mit Angabe der statistischen Methodik, einschließlich der Begründung der Fallzahl. Angabe des/der Statistikers/Statistikerin	**Versuchsplan:**<br>Es handelt sich um ein randomisiertes Placebo-kontrolliertes Versuchsdesign.<br>**Abhängige Variablen:**<br>*Primär:* Veränderung der kognitiven Leistung in einer validierten Lern- und Merkfähigkeitsaufgabe im Vergleich Baseline (T1) und nach Beendigung der Intervention (T2)<br>*Sekundär:* Folgende Veränderungen werden im Vergleich Baseline (T1) und nach Beendigung der Intervention (T2) geprüft:<br>1) Weitere kognitive Leistungen<br>2) periphere Biomarker (Polyamin-Konzentration im Blut, Entzündungsmarker und vaskuläre Prozesse)<br>**Auswertung:**<br>Bezüglich der Haupthypothese werden zunächst die 15 polyamin-einnehmenden Probanden mit den 15 Placebo einnehmenden Probanden verglichen.<br>Mit diesen Fallzahlen (n=15 Polyamin vs. n=15 Placebo) kann ein zweiseitiger t-test für unabhängige Stichproben (die jeweiligen Differenzen zwischen *baseline* und *follow up* zu einem Kognitionsscore) einen Unterschied mit 80% Power zeigen, wenn die Effektstärke 1,06 ist (zweiseitiges Signifikanzniveau: α=0,049). Da dies eine *Proof-of-Concept* Studie ist, dient die Effektstärke im Falle eines nichtsignifikanten Unterschieds der Fallzahlberechnung für eine umfangreichere Haupt-Studie.<br>Die Poweranalyse erfolgt konservativ für einen t-Test für Differenzen (*Follow-up*-Wert – *Baseline*-Wert) der Polyamin-Konzentrationen in den beiden Gruppen (Intervention und

	Placebo) auch wenn die Analyse mittels Kovarianzanalyse erfolgt.<br><br>Die Analyse der sekundären Fragestellungen erfolgt in dieser Vorstudie explorative.<br><br><u>**Randomisierung**</u><br><br>Es erfolgt eine stratifizierte Randomisierung nach Geschlecht und Alter (60-70, 70-80 Jahre).<br><br>**VORGESCHLAGENE STICHPROBENGRÖßE/POWER-KALKULATION**<br><br>Die vorgeschlagene Stichprobengröße wurde von der bereits existierenden Studie von Soda und Kollegen (2009b) abgeleitet, welche eine positive Wirkung von polyaminreicher Diät auf die körpereigenen Polyaminspiegel nachweisen konnte.<br><br>Die Studie wird biometrisch betreut durch Frau Dr. Grittner.
16.<br><br>a. Darlegung und ggf. Erläuterung der **Ein- und Ausschlusskriterien**	<u>**Einschlusskriterien:**</u><br><br>- kognitiv intakte ältere Menschen mit erhöhtem Demenzrisiko operationalisiert als:<br>• subjektiv empfundene Gedächtnisverschlechterung und diesbezügliche Sorgen, mitgeteilt durch die Person<br>• keine objektiven Gedächtnisschwierigkeiten (gemessen mittels geschlechts-, alters- und bildungs-adjustierten Normwerte)<br>• altersentsprechende Leistungen in anderen kognitiven Domänen<br>• keine Einschränkungen bei sonstigen Alltagsaktivitäten<br>• keine DSM-IV manifeste Demenz<br>- Alter: 60-80 Jahre<br>- Bestehende Krankenversicherung zur Abklärung möglicher Zufallsbefunde<br>- Einwilligungsfähigkeit<br><br><u>**Ausschlusskriterien:**</u><br><br>- schwere internistische oder psychiatrische Erkrankungen<br>- Malignome aktuell oder in der Anamnese<br>- neurologische Erkrankungen (z.B. Epilepsie auch bei positiver Familienanamnese, Migräne)<br>- schwere Arteriosklerose, fortgeschrittener Herz- oder Atemwegserkrankung, schwere Leber-, Nieren- oder Stoffwechselkrankheiten, unbehandelten Schilddrüsenfunktionsstörungen oder unbehandeltem Diabetes mellitus („Zuckerkrankheit“)<br>- Erkrankungen, die eine generelle Einschränkung der Aufmerksamkeitsleistung beinhalten<br>- Manifeste Demenz<br>- Gerinnungsstörungen, Marcumar Therapie, oder ASS-

	Therapie<br>- Drogen- / Medikamenten -/ Alkoholabhängigkeit<br>- bereits vorhandene Polyamin-Substitution<br>- bekannte Unverträglichkeit oder Allergien auf Weizenkeimlinge oder bekannte Glutenunverträglichkeit und Histaminintoleranz<br>- Hirntumore, territoriale Schlaganfälle (aus zerebralem MRT)<br><br>Auch Personen, die eine Klaustrophobie oder metallische Implantate oder Tattoos aufweisen, werden aufgrund der Nicht-Durchführbarkeit des MRTs ausgeschlossen.
b. **Teilnehmerinformation** (wer diese mündlich erteilt und Angabe, wie viel Zeit zwischen Aufklärung und Einwilligung verbleibt, ansonsten Verweis auf deren Inhalt als Anlage möglich)	siehe Teilnehmerinformation anbei
c. **Einwilligungserklärung** (Verweis auf deren Inhalt als Anlage möglich)	siehe Einwilligungserklärung anbei
d. Ggf. **Information und Einwilligung des gesetzlichen Vertreters** (ggf. auch Beschreibung des Verfahrens zur Einrichtung einer gerichtlichen Betreuung)	Entfällt, da nur gesunde Studienteilnehmer eingeschlossen werden, die selbst ihre Einwilligung erteilen können.
17. Maßnahmen zur Gewinnung von Studienteilnehmern (Aushang ?, Zeitungsannoncen? Etc.)	Die Studie wird in den Berliner Gedächtnissprechstunden beworben. Außerdem gibt es im Internet Informationen und Kontaktmöglichkeiten zur Studie, z.B. auf den Internetseiten der Klinik für Neurologie der Charité und auf www.probandeninfo.de. Je nach Rekrutierungserfolg wird es zusätzlich Annoncen in Berliner Zeitungen geben. Die Anwerbung erfolgt weiterhin über Aushänge in Volkshochschulen oder Seniorenvereinen, Kontaktierung der BANA (Berliner Modell für nachberufliche Aktivitäten, in denen Menschen ab 45 Zugang zu universitärer Weiterbildung erhalten).
18. Ggf.: **Grund für die Einbeziehung und Darlegung des therapeutischen Nutzens für Personen, die minderjährig und/oder nicht einwilligungsfähig sind.**	entfällt , s. o.
19. Beziehung zwischen Studienteilnehmer und Studienarzt/-ärztin (Ist der Studienarzt zugleich der behandelnde Arzt?)	Bei Rekrutierung über nicht-eigene Gedächtnissprech-stunden sind die Studienärzte nicht-behandelnde Ärzte (behandelnder Arzt = Arzt der jeweiligen Gedächtnissprechstunde). Bei Rekrutierung innerhalb der Gedächtnissprechstunde Mitte wird der Studienarzt der nicht-behandelnde Arzt sein.<br><br>Die kognitiven Leistungstests werden von den

	wissenschaftlichen Mitarbeitern der AG Flöel durchgeführt. Die MRT-Untersuchungen werden unter Leitung von Prof. John-Dylan Haynes auf dem Campus Charité Mitte durchgeführt. Neuroradiologische Beurteilung der MRT Aufnahmen erfolgt durch PD Dr. Jochen Fiebach.<br><br>Die Bestimmung der körpereigenen Polyamin-Konzentration wird im Labor von Prof. Stephan J. Sigrist durchgeführt.
20. Erklärung zur Einbeziehung möglicherweise vom Sponsor oder Studienarzt abhängiger Personen	Die Studienteilnehmer, die in die Untersuchung eingeschlossen werden, sind weder vom Studienarzt noch vom einen Sponsor abhängig.
21. Maßnahmen, die eine Feststellung zulassen, ob ein Studienteilnehmer an mehreren Studien zugleich oder vor Ablauf einer in der vorangegangenen Studie festgelegten Frist teilnimmt.	Beim Eingangsscreening (mündlich oder fernmündlich) wird nach vorherigen/gleichzeitigen anderen Studienteilnahmen gefragt, und wenn ja bei welcher/n.
22. Ggf.: Honorierung bzw. Kostenerstattung der Studienteilnehmer (Höhe, wofür soll gezahlt werden ?)	Am Ende der Studie wird eine Aufwandsentschädigung von 85 Euro gezahlt (einmalige Pauschale für Zeitaufwand und Fahrtkosten)
23. Ggf.: Plan für die Weiterbehandlung und medizinische Betreuung der betroffenen Personen nach dem Ende der Studie	Werden Teilnehmer aus Gedächtnissprechstunden rekrutiert, werden sie im Rahmen der jeweiligen Sprechstunde weiter betreut.
24. Ggf.: Versicherung der Studienteilnehmer (Versicherungsbestätigung und Versicherungsbedingungen, Versicherer, Versicherungsumfang, Versicherungsdauer)	Entfällt, die Studienärzte werden über die Betriebshaftpflichtversicherung der Charité versichert.
25. Ggf.: Dokumentationsverfahren (Verweis auf CRF-Bögen möglich)	Es wird eine Studienakte geführt.<br><br>**Folgende personenbezogene Daten werden erfasst:**<br>- Alter, Geschlecht, Geburtsdatum, Bildungsgrad, Adresse, Daten zur Gesundheit/Erkrankung<br><br>**Datenkategorien:**<br>Studiendaten<br><br>**Erhebungsart:**<br>- Daten werden sowohl über *paper pencil* als auch über computerunterstützte Tests erfasst<br>- Die Tests enthalten keine Klarnamen sondern das für jede/n Teilnehmer/in festgelegte Pseudonym (siehe Punkt 28).<br>- Alle Daten werden in eine/mehrere digitale Datenmatrix/Datenmatrizen in pseudonymisierter Form übertragen und gespeichert<br><br>**Umgang mit Daten und Proben:**<br>Blutproben:<br>- werden pseudonymisiert, d.h. mit Code (siehe Punkt 28), Alter und Geschlecht (letzteres für Referenzwerte)

	versehen und zum Zweck der Studie an die externen Labore (s. Punkt 30) versendet<br><br>MRT-Daten<br><br>- werden pseudonymisiert zur Befundung an einen Neuroradiologen übermittelt<br>- bei auffälligen Befunden wird der Proband über die Teilnehmeridentifizierungsliste (siehe Punkt 28) ermittelt und ggf. darüber informiert<br><br>Testergebnisse<br><br>- die Teilnehmer haben das Recht Auskunft zu verlangen; das kann mündlich oder in Form von Ausdrucken erfolgen (auch hier kann der Proband ausschließlich über die Teilnehmeridentifizierungsliste den Testergebnissen zugeordnet werden)<br><br>**Aufbewahrung:**<br><br>Personenbezogene Daten:<br><br>- werden entsprechend dem Nachweis guter wissenschaftlicher Praxis für einen Zeitraum von 10 Jahren gespeichert, danach gelöscht und anschließend ggf. nur in rein anonymer Form weiter verwendet<br><br>Blutproben<br><br>- werden für mögliche Nachuntersuchungen über einen Zeitraum von höchstens 10 Jahre nach Studienende in den jeweiligen Analyse-Laboren gelagert und danach vernichtet<br><br>**Zugang zu den Daten und Proben:**<br><br>- zu den personenbezogenen Daten werden nur die an der Studie beteiligten Mitarbeiter der Arbeitsgruppe Flöel Zugang haben<br>- alle Mitarbeiter der Arbeitsgruppe Flöel unterliegen der Schweigepflicht<br>- Proben werden ausschließlich pseudonymisiert weiter verwendet
26. Ggf.: Beschreibung, wie der Gesundheitszustand gesunder betroffener Personen dokumentiert werden soll	Die Studienteilnehmer werden nach ihrem Gesundheitsstatus hinsichtlich bestimmter Kriterien (siehe Punkt 16; Ausschlusskriterien) befragt.
27. Ggf.: Methoden, unerwünschte Ereignisse festzustellen, zu dokumentieren und mitzuteilen (wann, von wem und wie ??)	Während der Untersuchung werden die Teilnehmer im direkten Kontakt mit den betreuenden wissenschaftlichen Mitarbeitern und Neurologen stehen. Für die Zeit nach Abschluss der Untersuchung und für den Fall des Auftretens etwaiger Nebenwirkungen wird eine Telefonnummer zur Verfügung gestellt, unter der immer ein Neurologe erreichbar ist.<br><br>Während der 3-monatigen Interventionsphase wird ein

	Telefongespräch geplant, um die Compliance und Verträglichkeit der Interventionen zu dokumentieren. Dabei werden auch unerwünschte Ereignisse erfragt und dokumentiert.<br><br>Bei den gesunden Studienteilnehmern können sogenannte Zufallsbefunde auftreten (z. B. bisher unbekannte hirnstrukturelle Veränderungen); daher werden alle MRTs radiologisch von einem Facharzt begutachtet.

28. Vorgehen zum Schutz der Geheimhaltung der gespeicherten Daten, Dokumente und ggf. Proben, Darlegung der Verschlüsselung der Daten von Studienteilnehmern *(bitte nicht Initialen und Geburtsdatum als Codierungsschema verwenden!)*	Alle personenbezogenen Daten werden nach Einschluss der/des Teilnehmer/in in pseudonymisierter Form erhoben und gespeichert. In Papierform erhobene studienbezogene Daten (Tests, Fragebogen) werden in einem verschließbaren Schrank in einem Büro innerhalb der Neurologie aufbewahrt. Nach Eingabe der Daten in elektronischer Form werden diese auf einem Passwort geschützten Computer der Arbeitsgruppe gespeichert.<br><br>Es wird folgender Code vergeben: Buchstaben „SMART-PRE“, plus dreistelliger Zahl für den jeweiligen Studienteilnehmer (z.B. „001“ für den ersten Teilnehmer), plus Code für die jeweilige Messung („-1“ für Prä-Intervention bzw. T1, „-2“ für Post-Intervention bzw. T2), z. B.: „SMART-PRE-001-1“.<br><br>Die jeweiligen Identifizierungscodes der Studienteilnehmer werden in einer separaten Datei (Teilnehmeridentifikationsliste) erfasst und auf dem Server der AG gespeichert, der hierfür ein gesondertes Datenschutzkonzept hat. Das Anlegen einer Datei mit personenbezogenen Informationen wird dem Datenschutzbeauftragten der Charité gemeldet.<br><br>Die Einwilligungserklärungen der Studienteilnehmer werden in Papierform in einem verschließbaren Schrank der AG Flöel aufbewahrt.<br><br>**Zugriffsrechte**<br><br>- während und nach der Studie (siehe Punkt 25 und 29)<br><br>**Verfahren und Fristen für Löschung und Sperrung der Daten:**<br><br>- Die personenbezogenen Daten werden entsprechend dem Nachweis guter wissenschaftlicher Praxis für einen Zeitraum von 10 Jahren gespeichert, danach gelöscht und **anschließend ggf. nur in rein anonymer Form weiter verwendet**.<br><br>- Die Teilnehmer können Ihr Einverständnis zur Teilnahme an der Studie jederzeit widerrufen. Sie können auch jederzeit die Weiterverarbeitung der erhobenen Daten widersprechen und ihre Löschung bzw. Vernichtung verlangen. Alle Daten werden dann unmittelbar vernichtet.<br><br>- Es findet eine manuelle Löschung der Daten auf den PCs

	statt. Weiterhin werden in Papierform vorliegende Daten (Fragebogen, Testungen) per Schredder vernichtet. *Back-up*-DVDs werden ebenfalls zerstört.
29. Erklärung zur Einhaltung des Datenschutzes	Die im Rahmen dieser Studie personenbezogene Daten/Angaben werden durch die Studienärzte/-mitarbeiter erhoben und pseudonymisiert auf elektronischen Datenträgern aufgezeichnet und verarbeitet.<br><br>Sämtliche Mitarbeiter der AG Flöel unterzeichnen bei Dienstantritt das interne Merkblatt zum Datenschutz, welches eine Erklärung zum Datenschutz enthält und alle Mitarbeiter unterliegen der Schweigepflicht.<br><br>Die Datei mit dem Teilnehmeridentifizierungscode ist auf dem Server der Charité Laufwerk S passwortgeschützt gespeichert. Darauf haben nur die Studienleitung und von ihr beauftragte Mitarbeiter Zugriff.<br>Die Zugriffsberechtigung wird durch Frau Prof. Flöel erteilt und erlischt nach Ende der Studienmitarbeit.<br><br>Eine Datenübermittlung an andere Stellen - innerhalb oder außerhalb des öffentlichen Bereiches - ist nicht vorgesehen. Die Weitergabe der Daten an die Kooperationspartner erfolgt ausschließlich in pseudonymisierter Form.<br><br>Die Dateien werden so lange aufbewahrt, wie dies im Rahmen von Studien gesetzlich vorgeschrieben und entsprechend des Nachweises guter wissenschaftlicher Praxis geregelt ist (10 Jahre) und anschließend gelöscht. Einzelne Datensätze können auf Wunsch des Studienteilnehmers bereits im Studienverlauf gelöscht werden (z.B. bei Rückzug der Einwilligung zur Verarbeitung der personenbezogenen Daten). Unter Löschung werden die endgültige Löschung des jeweiligen einzelnen Datensatzes und das Entfernen etwaiger Sicherheitskopien verstanden.<br><br>Die Teilnehmer haben das Recht Auskunft über ihre Ergebnisse zu verlangen. Das kann mündlich oder in Form von Ausdrucken erfolgen.<br><br>Alle Mitarbeiter unterzeichnen bei Dienstantritt ein Formblatt, wodurch sie sich zur Befolgung der abteilungsinternen Richtlinien, insbesondere in Bezug auf vertrauliche Informationen, Schweigepflicht und Schutz personenbezogener Daten verpflichten (s.o.). Insbesondere wird die unbefugte Weitergabe von Daten an Dritte und das Verbringen schutzwürdiger Daten nach außen verboten.
30. Namen und Anschriften der Einrichtungen, die als Studienzentrum oder Studienlabor in die Studie eingebunden sind, sowie der Studienleiter und die Studienärzte	**Studienzentrum:**<br>NeuroCureClinical Research Center/<br>Klinik und Poliklinik für Neurologie, Charité Universitätsmedizin Berlin, Charitéplatz 1, 10117 Berlin<br>Die Studie wird in Berlin an der Charité stattfinden.

	**Studienleitung:**<br>Prof. Dr. med. Agnes Flöel<br><br>**Co-Studienleitung**<br>Dr. phil. nat. Dipl. Psych. Miranka Wirth<br><br>**Studienärzte:**<br>Prof. Dr. med. Agnes Flöel<br>Dr. med. J. List<br><br>**BMBF-Kooperationspartner** (Die Studie wird in Berlin an der Charité stattfinden)<br><br>Prof. Stephan J. Sigrist, Ph.D.<br>Freie Universität Berlin, Institut für Biologie/Genetik<br>Takustrasse 6, 14195 Berlin und<br>CharitéCrossOver (CCO), Virchowweg 6<br>10117 Berlin<br><br>**Bereitstellung der Kapseln:**<br>Prof. Dr.rer.nat. Frank Madeo<br>Alterung & Zelltod - Labor<br>Institut für Molekulare Biowissenschaften<br>Humboldtstrasse 50/EG<br>8010 Graz, Austria<br><br>**Studienlabore:**<br>Polyamin-Untersuchung<br>Prof. Stephan J. Sigrist, Ph.D.<br>Freie Universität Berlin, Institut für Biologie/Genetik<br>Takustrasse 6, 14195 Berlin (Germany) and<br>CharitéCrossOver (CCO), Virchowweg 6<br>10117 Berlin<br>Blutuntersuchung<br>Institute für Medizinische Diagnostik MVZ GbR, Laboratoriumsmedizin Mikrobiologie Transfusionsmedizin, Nicolaistr. 22, 12247 Berlin-Steglitz<br><br>**Kooperationspartner:**<br>Prof. John-Dylan Haynes (im Rahmen der Erhebung struktureller und funktioneller MRT-Daten); Charité – Universitätsmedizin Berlin, Bernstein Center for Computational Neuroscience, Philippstrasse 13, Haus 6, 10117 Berlin
31. Angaben zur Eignung der Prüfstelle, insbesondere zur Angemessenheit der dort vorhandenen Mittel und Einrichtungen sowie des zur Durchführung der klinischen Prüfung zur Verfügung	Die Studie wird vom BMBF finanziert. Die Studienärzte sind in der Charité angestellt. Untersuchungsräume und Laborplätze sind ausreichend vorhanden, ebenso das zur Testung benötigte Material. Über das Projekt werden naturwissenschaftliche/medizinische Doktoranden eingestellt, die bei der Durchführung und Analyse der

stehenden Personals und zu Erfahrungen in der Durchführung ähnlicher Studien	Untersuchungen helfen. Studienleitung und Studienärzte verfügen bereits über umfassende Erfahrungen in der Durchführung ähnlicher Studien.
32. Vereinbarung über den Zugang des Prüfers/Hauptprüfers/Leiter der klinischen Prüfung, zu den Daten und den Grundsätzen über die Publikation	Die Studiendaten werden durch die Studienleitung publiziert. Die Studienergebnisse werden in anonymer Form, die keinen Rückschluss auf die teilnehmende Person zulassen, veröffentlicht.
33. Angaben zur Finanzierung der Studie:<br>Finanzierungsquelle (Name und Sitz)	Bundesministerium für Bildung und Forschung (BMBF)<br>Hannoversche Straße 28-30, 10115 Berlin

Name und Unterschrift des/der Antragstellers:

Ich versichere hiermit, dass die in diesem Antrag gegebenen Informationen richtig sind. Ich bin der Auffassung, dass es möglich ist, die o.g. Studie in Übereinstimmung mit dem Protokoll, den nationalen Rechtsvorschriften durchzuführen.

Mir ist bekannt, dass ich gemäß §19 Berliner Datenschutzgesetz (BlnDSG) verpflichtet bin, für automatisierte Verarbeitungen personenbezogener und personenbeziehbarer Daten eine Datei- und Verfahrensbeschreibung zu erstellen und diese gemäß §19a dem behördlichen Datenschutzbeauftragten der Charité zur Verfügung stellen muss. Ich bin darüber informiert, dass wenn es sich um ein Verfahren handelt, mit dem Daten verarbeitet werden, die einem Berufsgeheimnis (z.B. ärztliche Schweigepflicht) unterliegen, ich gemäß §5 BlnDSG vor dem Einsatz dieses Verfahrens eine Vorabkontrolle durch den behördlichen Datenschutzbeauftragten der Charité veranlassen muss und ich das Verfahren erst bei positivem Prüfergebnis anwenden darf.

Name: Flöel
Vorname: Agnes
Adresse: Charité Universitätsmedizin Berlin, Klinik und Poliklinik für Neurologie, NeuroCure Clinical Research Center, Campus Mitte, Charitéplatz 1, 10117 Berlin
Position: Univ.-Prof., OÄ

Datum:

Unterschrift:

Anlagen
- Teilnehmerinformation Version 1.0 vom 29.07.2015
- Einwilligungserklärung Version 1.0 vom 29.07.2015
- Bestätigung der Betriebshaftpflichtversicherung

Literaturverzeichnis:

Ali MA, Poortvliet E, Strömberg R, Yngve A (2011) Polyamines: total daily intake in adolescents compared to the intake estimated from the Swedish Nutrition Recommendations Objectified (SNO). Food & Nutrition Research 55:10.3402/fnr.v3455i3400.5455.

Baddeley AD, Wilson B, Watts F (1995) Handbook of Memory Disorders: Chichester, W.Sussex: John Wiley & Sons Ltd.

Bardocz S, Duguid TJ, Brown DS, Grant G, Pusztai A, White A, Ralph A (1995) The importance of dietary polyamines in cell regeneration and growth. Br J Nutr 73:819-828.

Bell MR, Belarde JA, Johnson HF, Aizenman CD (2011) A neuroprotective role for polyamines in a Xenopus tadpole model of epilepsy. Nature neuroscience 14:505-512.

Bickel H (2000) Demenzsyndrom und Alzheimer Krankheit: Eine Schätzung des Krankenbestandes und der jährlichen Neuerkrankungen in Deutschland. Gesundheitswesen 62:211-218.

Brown BM, Peiffer JJ, Taddei K, Lui JK, Laws SM, Gupta VB, Taddei T, Ward VK, Rodrigues MA, Burnham S, Rainey-Smith SR, Villemagne VL, Bush A, Ellis KA, Masters CL, Ames D, Macaulay SL, Szoeke C, Rowe CC, Martins RN (2012) Physical activity and amyloid-beta plasma and brain levels: results from the Australian Imaging, Biomarkers and Lifestyle Study of Ageing. Molecular psychiatry.

Chen GG, Fiori LM, Moquin L, Gratton A, Mamer O, Mechawar N, Turecki G (2010) Evidence of altered polyamine concentrations in cerebral cortex of suicide completers. Neuropsychopharmacology : official publication of the American College of Neuropsychopharmacology 35:1477-1484.

Duzel E, Schutze H, Yonelinas AP, Heinze HJ (2011) Functional phenotyping of successful aging in long-term memory: Preserved performance in the absence of neural compensation. Hippocampus 21:803-814.

Eisenberg T et al. (2009) Induction of autophagy by spermidine promotes longevity. Nature cell biology 11:1305-1314.

Ferchmin PA, Perez D, Biello M (2000) Spermine is neuroprotective against anoxia and N-methyl-D-aspartate in hippocampal slices. Brain Res 859:273-279.

Ficker E, Taglialatela M, Wible BA, Henley CM, Brown AM (1994) Spermine and spermidine as gating molecules for inward rectifier K+ channels. Science 266:1068-1072.

Folstein M, Folstein S, McHugh P (1975) “Mini-mental state”: a practical method for grading the cognitive state of patients for the clinicians. Journal of Psychiatric Research 12:189-198.

Gerner EW, Meyskens FL, Jr. (2004) Polyamines and cancer: old molecules, new understanding. Nat Rev Cancer 4:781-792.

Gupta VK, Scheunemann L, Eisenberg T, Mertel S, Bhukel A, Koemans TS, Kramer JM, Liu KS, Schroeder S, Stunnenberg HG, Sinner F, Magnes C, Pieber TR, Dipt S, Fiala A, Schenck A, Schwaerzel M, Madeo F, Sigrist SJ (2013) Restoring polyamines protects from age-induced memory impairment in an autophagy-dependent manner. Nature neuroscience 16:1453-1460.

Härting C, Markowitsch H, Neufeld H, Calabrese P, Dejerine J, Deisinger K (2000) Wechsler Gedächtnistests - revidierte Fassung (WMS-R). Bern: Huber.

Ibe S, Kumada K, Yoshida K, Otobe K (2013) Natto (fermented soybean) extract extends the adult lifespan of Caenorhabditis elegans. Biosci Biotechnol Biochem 77:392-394.

Ishida S, Sarada M, Seki H, McGirr L, Lau A, Morishita K (2013) Genotoxicity and subchronic oral toxicity of L-ornithine monohydrochloride. Regul Toxicol Pharmacol 67:360-371.

Jessen F, Wolfsgruber S, Wiese B, Bickel H, Mosch E, Kaduszkiewicz H, Pentzek M, Riedel-Heller SG, Luck T, Fuchs A, Weyerer S, Werle J, van den Bussche H, Scherer M, Maier W, Wagner M, German Study on Aging C, Dementia in Primary Care P (2014) AD dementia risk in late MCI, in early MCI, and in subjective memory impairment. Alzheimers Dement 10:76-83.

Kessels RP, van Zandvoort MJ, Postma A, Kappelle LJ, de Haan EH (2000) The Corsi Block-Tapping Task: standardization and normative data. Applied neuropsychology 7:252-258.

Kohlmeier L (1995) Future of dietary exposure assessment. The American Journal of Clinical Nutrition 61:702S-709S-702S-709S.

Kramer DL, Diegelman P, Jell J, Vujcic S, Merali S, Porter CW (2008) Polyamine acetylation modulates polyamine metabolic flux, a prelude to broader metabolic consequences. The Journal of biological chemistry 283:4241-4251.
Lezak MD (2004) Neuropsychological assessment, 4 Edition: New York, Oxford: Oxford University Press.
Minois N, Carmona-Gutierrez D, Madeo F (2011) Polyamines in aging and disease. Aging 3:716-732.
Morrison LD, Becker L, Ang LC, Kish SJ (1995) Polyamines in human brain: regional distribution and influence of aging. Journal of neurochemistry 65:636-642.
Nishibori N, Fujihara S, Akatuki T (2007) Amounts of polyamines in foods in Japan and intake by Japanese. Food Chemistry 100:491-497.
Okamoto A, Sugi E, Koizumi Y, Yanagida F, Udaka S (1997) Polyamine content of ordinary foodstuffs and various fermented foods. Biosci Biotechnol Biochem 61:1582-1584.
Oliver D, Baukrowitz T, Fakler B (2000) Polyamines as gating molecules of inward-rectifier K+ channels. Eur J Biochem 267:5824-5829.
Park MH, Igarashi K (2013) Polyamines and Their Metabolites as Diagnostic Markers of Human Diseases. Biomol Ther (Seoul) 21:1-9.
Pegg AE (2009) Mammalian polyamine metabolism and function. IUBMB Life 61:880-894.
Pegg AE, Feith DJ (2007) Polyamines and neoplastic growth. Biochem Soc Trans 35:295-299.
Pegg AE, Wang X (2009) Mouse models to investigate the function of spermine. Commun Integr Biol 2:271-274.
Pucciarelli S, Moreschini B, Micozzi D, De Fronzo GS, Carpi FM, Polzonetti V, Vincenzetti S, Mignini F, Napolioni V (2012) Spermidine and spermine are enriched in whole blood of nona/centenarians. Rejuvenation Res 15:590-595.
Ralph A, Englyst K, Bardócz S (1999) Polyamine content of the human diet. In: Polyamines in Health and Nutrition (Bardócz S, White A, eds), pp 123-137. Londin, UK: Kluwer Academic Publishers.
Ransom RW, Stec NL (1988) Cooperative Modulation of [3H]MK-801 Binding to the N-Methyl-d-Aspartate Receptor-Ion Channel Complex by l-Glutamate, Glycine, and Polyamines. Journal of neurochemistry 51:830-836.
Reitan RM, Herring S (1985) A short screening device for identification of cerebral dysfunction in children. Journal of Clinical Psychology 41:643-650.
Russell D, Snyder SH (1968) Amine synthesis in rapidly growing tissues: ornithine decarboxylase activity in regenerating rat liver, chick embryo, and various tumors. Proceedings of the National Academy of Sciences 60:1420-1427.
Schaeffer V, Lavenir I, Ozcelik S, Tolnay M, Winkler DT, Goedert M (2012) Stimulation of autophagy reduces neurodegeneration in a mouse model of human tauopathy. Brain 135:2169-2177.
Soda K, Dobashi Y, Kano Y, Tsujinaka S, Konishi F (2009a) Polyamine-rich food decreases age-associated pathology and mortality in aged mice. Experimental gerontology 44:727-732.
Soda K, Kano Y, Chiba F, Koizumi K, Miyaki Y (2013) Increased polyamine intake inhibits age-associated alteration in global DNA methylation and 1,2-dimethylhydrazine-induced tumorigenesis. PLoS One 8:e64357.
Soda K, Kano Y, Sakuragi M, Takao K, Lefor A, Konishi F (2009b) Long-term oral polyamine intake increases blood polyamine concentrations. Journal of nutritional science and vitaminology 55:361-366.
Stark SM, Yassa MA, Lacy JW, Stark CE (2013) A task to assess behavioral pattern separation (BPS) in humans: Data from healthy aging and mild cognitive impairment. Neuropsychologia 51:2442-2449.
Tersey SA, Colvin SC, Maier B, Mirmira RG (2014) Protective effects of polyamine depletion in mouse models of type 1 diabetes: implications for therapy. Amino Acids 46:633-642.
Tiboldi A, Lentini A, Provenzano B, Tabolacci C, Hoger H, Beninati S, Lubec G (2012) Hippocampal polyamine levels and transglutaminase activity are paralleling spatial memory retrieval in the C57BL/6J mouse. Hippocampus 22:1068-1074.
Til HP, Falke HE, Prinsen MK, Willems MI (1997) Acute and subacute toxicity of tyramine, spermidine, spermine, putrescine and cadaverine in rats. Food Chem Toxicol 35:337-348.
Trenerry MR, Crosson B, DeBoe J, Leber WR (1989) Stroop Neuropsychological Screening Test manual. In. Odessa, FL: Psychological Assessment Resources.

Vivo M, de Vera N, Cortes R, Mengod G, Camon L, Martinez E (2001) Polyamines in the basal ganglia of human brain. Influence of aging and degenerative movement disorders. Neuroscience letters 304:107-111.
Watson D, Clark LA, Carey G (1988) Positive and negative affectivity and their relation to anxiety and depressive disorders. J Abnorm Psychol 97:346-353.
Wechsler D (1997a) Wechsler Adult Intelligence Scale-III. San Antonio, TX: The Psychological Corporation.
Wechsler D (1997b) Wechsler Memory Scale - Third edition. Manual. San Antonio, TX: The Psychological Corperation.
Welsh PA, Sass-Kuhn S, Prakashagowda C, McCloskey D, Feith D (2012) Spermine synthase overexpression in vivo does not increase susceptibility to DMBA/TPA skin carcinogenesis or Min-Apc intestinal tumorigenesis. Cancer Biol Ther 13:358-368.
Yan DH, Nishimura K, Yoshida K, Nakahira K, Ehara T, Igarashi K, Ishihara K (2005) Different intracellular polyamine concentrations underlie the difference in the inward rectifier K(+) currents in atria and ventricles of the guinea-pig heart. J Physiol 563:713-724.
Yao Jk, Stanley JA, Reddy RD, Keshavan MS, Pettegrew JW (2002) Correlations between peripheral polyunsaturated fatty acid content and in vivo membrane phospholipid metabolites. Biological Psychiatry 52:823-830.
Yesavage JA, Brink TL, Rose TL, Lum O, Huang V, Adey M, Leirer VO (1982) Development and validation of a geriatric depression screening scale: a preliminary report. Journal of psychiatric research 17:37-49.
Zimmermann P, Fimm B (1993) Testbatterie zur Aufmerksamkeitsprü fung (TAP). Freiburg: Psytes.
Zoumas-Morse C, Rock CL, Quintana EL, Neuhouser ML, Gerner EW, Meyskens FLJ (2007) Development of a Polyamine Database for Assessing Dietary Intake. Journal of the American Dietetic Association 107:1024-1027.
